# Multiple simulated spaceflight stressors impact cardiac fibrosis, calcium dynamics, immune function, cytokines and gene variants in rat, *Rattus norvegicus*

**DOI:** 10.1371/journal.pone.0357485

**Published:** 2026-09-30

**Authors:** Marek Lenarczyk, Amy Kronenberg, Andy Kwok, Alexander Borg, Ammar J. Alsheikh, Angela J. Mathison, Michael T. Zimmermann, Samuel B. R. Lawton, Hershel Raff, Jeffrey Willey, Richard A. Komorowski, Raul Urrutia, John E. Baker

**Affiliations:** 1 Radiation Biosciences Laboratory, Medical College of Wisconsin, Milwaukee, Wisconsin, United States of America; 2 BioEngineering and BioMedical Sciences, Lawrence Berkeley National Laboratory, Berkeley, California, United States of America; 3 Department of Radiation Oncology, Wake Forest School of Medicine, Winston Salem, North Carolina, United States of America; 4 Department of Physiology, Medical College of Wisconsin; Milwaukee, Wisconsin, United States of America; 5 Mellowes Center, Medical College of Wisconsin, Milwaukee, Wisconsin, United States of America; 6 Department of Surgery, Medical College of Wisconsin, Milwaukee, Wisconsin, United States of America; 7 Data Science Institute, Medical College of Wisconsin, Milwaukee, Wisconsin, United States of America; 8 Department of Medicine and Cardiovascular Center, Medical College of Wisconsin, Milwaukee, Wisconsin, United States of America; 9 Department of Pathology, Medical College of Wisconsin, Milwaukee, Wisconsin, United States of America; Royal College of Surgeons in Ireland, IRELAND

## Abstract

Space radiation and microgravity pose significant health risks for astronauts conducting deep space exploration. The impact of galactic cosmic radiation (GCR) and weightlessness alone, and their combined effects on cardiac disease, the immune system, cytokines and calcium dynamics should be understood before undertaking such missions. Male WAG/RijCmcr rats, *Rattus norvegicus,* 8–9 months old were hind limb unloaded (HLU) to simulate exposure to microgravity for 5 days before, during, and 25 days after whole-body exposure to a simplified GCR simulation (simGCRsim) dose of 1.5 Gy that was administered rapidly. Other rats maintained normal weight-bearing. Exposure to 1.5 Gy of simGCRsim with or without HLU increased cardiac perivascular collagen content, the primary endpoint, compared to sham-irradiated no HLU rats after 270 days. The extent of cardiac fibrosis present in the two irradiated groups could not be distinguished, indicating no interaction between the two space flight stressors. A series of secondary endpoints were also measured. Sham irradiation with HLU increased blood lymphocytes but not myeloid cells compared with non-HLU, an outcome that was inhibited by simGCRsim with HLU, indicating an interaction between spaceflight stressors at this high dose-rate. HLU alone decreased multiple circulating cytokines and depleted CD56^+^ cells in the spleen. Notably, simGCRsim and HLU alone and in combination did not disrupt calcium homeostasis. An exposure to 1.5 Gy simGCRsim no HLU revealed distinct genomic alterations in circulating nucleated blood cells as compared with sham-irradiated subjects no HLU, and identified mutations, frameshifts, and deletions in genes that contribute to human disease in the irradiated cohort. A limited follow-on study involved exposure to 0.75 Gy simGCRsim with no HLU with appropriate controls. This radiation dose, a dose relevant to human missions to Mars, caused cardiac perivascular fibrosis at an extended follow up period of after 360 days. Taken together, our findings show exposure to simGCRsim, representing the composition of GCR behind spacecraft shielding, caused cardiac perivascular fibrosis and gene variants. Simulated microgravity (HLU) alone engaged the immune system and decreased cytokine production. We conclude that exposure of rats to simGCRsim and/or HLU results in persistent health effects with limited interactions between the two spaceflight stressors.

## Introduction

Space radiation and microgravity pose two significant health risks for astronauts conducting deep space exploration missions. Humans have evolved with minimal exposure to galactic cosmic radiation (GCR) and with the constant downward force of gravity. Extended duration exploration missions to the Earth’s Moon and the planet Mars are actively being prepared. During these missions, astronauts will be exposed to GCR once outside the Earth’s magnetosphere, and the force of gravity will decrease as the distance increases from the surface of the Earth to the Moon or Mars. Humans will encounter reduced gravity on lunar and Martian surfaces. The extent to which human health will be adversely affected by these spaceflight stressors during and after the completion of exploration missions is a major unknown and a concern to NASA and other space agencies.

Space radiation environments include low to moderately energetic protons and alpha particles from solar particle events (SPE), as well as GCR, which is composed of higher energy electrons and positrons (2%), protons (85%), helium nuclei (12%), and heavier high-energy and high-charge ions (HZE; 1%, [1]. During exploratory missions, astronauts will be continuously exposed to GCR and less predictably and sporadically to SPE. Our initial studies determined the impact of space radiation on the risks for degenerative long-term cardiovascular disease at doses that are mission-relevant for future human spaceflight beyond low earth orbit [2]. Exposure of male WAG/RijCmcr rats at 6 months of age at a ground-based charged particle accelerator with three rapidly switched ion beams representative of components of GCR (protons, silicon ions, and iron ions) caused cardiac vascular pathology with an apparent threshold dose for cardiac perivascular fibrosis over the time course studied [2]. Fibrosis results from excess deposition of the extracellular matrix, primarily collagen types I and III. NASA recently developed the simplified GCR simulator (simGCRsim) at the NASA Space Radiation Laboratory (NSRL) at Brookhaven National Laboratory (BNL). The simGCRsim generates a spectrum of six ion beams (Table 2) that approximate the primary and secondary GCR field experienced at human organ locations within a deep space vehicle and low energy protons present in SPE [3]. This five-ion, six-beam simGCRsim is better at representing the space radiation environment than the three ion beams of protons, silicon, and iron [2] in our previous study, and can be used as a standard radiation stimulus to allow comparisons of space radiation studies between laboratories.

Exposure to microgravity affects human health. In astronauts, bone loss occurs and blood calcium levels are still decreased up to 1 year after return to the Earth [4]. Microgravity by dry immersion exposure in humans for 21 days results in the expansion of T cells that persists upon re-exposure to normal gravity for 7 days [5]. During 6 months of spaceflight, microgravity as well as other spaceflight stressors suppress the immune system [6]. Cytokines are secreted signaling proteins that activate distinct cell signaling and the immune response. These small proteins (~5–25 kDa) typically function by interacting with specific cytokine receptors on target cell surfaces. Studies of cosmonauts and astronauts living in microgravity during low earth orbit showed no change in circulating cytokines 30 days after returning to 1g [7–9].

Hind limb unloading (HLU) in rats causes a cephalad fluid shift and reduced mobility as reported during spaceflight in astronauts [10]. HLU is accepted by NASA as a rodent model of microgravity. Reloading studies in mice have been conducted up to 49 days after 30 days of HLU [10], and others have examined responses up to 270 days after 7 days of HLU [11,12]. In humans exposed to microgravity and rodents exposed to hind limb unloading, the cephalad redistribution of blood volume with the accompanying decrease in pressure gradient should decrease kidney perfusion. The kidney is mainly responsible for regulating calcium dynamics. We reasoned that the 270-day period needed for perivascular cardiac fibrosis to develop in WAG/RijCmcr rats after exposure to space-relevant doses of radiation [2] would be sufficient to detect the engagement of circulating immune cells, changes in cytokines, and biomarkers of altered calcium metabolism caused by HLU.

Changes in the sequence of DNA can trigger specific molecular alterations that can influence cellular physiology and may contribute to changes in higher order functions. Low- and high-linear energy transfer (LET) radiation are well known to affect the DNA structure in cells by inducing single or double-strand breaks along with base damages and complex lesions [13]. DNA in affected cells can either be repaired and function normally, be mis-repaired with permanent changes in the DNA sequence resulting in a gene variant that may function abnormally, or unrepaired or misrepaired changes that can lead to cell death. Aging can result in somatic mutations in DNA that drive clonal expansion of blood cells to cause clonal hematopoiesis of indeterminate potential (CHIP) that contributes to increased risk for cardiovascular disease [14]. We reasoned that as age and radiation are independent risk factors for the development of cardiovascular disease, whole genome sequencing of nucleated peripheral blood from mature rats of astronaut age exposed to simGCRsim would reveal gene variants that may contribute to CHIP.

The interaction between simulated weightlessness and space radiation on cardiovascular disease needs to be understood before undertaking extended deep space exploration missions. Until recently, combining the spaceflight hazard of reduced weight bearing *via* HLU in rats of any strain with simGCRsim in conscious animals has not been possible. We created a model protocol with rats simultaneously exposed to HLU and simGCRsim followed out for an extended period after the cessation of spaceflight stressors [15,16]. We tested the central hypothesis that exposure to HLU and space relevant doses of simGCRsim potentiate risk for chronic cellular dysfunction in multiple biological systems using a rat model with no pre-existing disease and that is sensitive to radiation-induced cardiac pathology. Our objective was to measure risk for cardiac disease, alterations of the immune system, cytokines, calcium metabolism, and gene variants. Understanding these risks is a priority based on both the likelihood of occurrence and the severity of their impact on human health for exploration missions.

## Materials and methods

### Study design

This study was a controlled laboratory experiment to evaluate whether the spaceflight stressors GCR and simulated weightlessness act synergistically on the male WAG/RijCmcr rat. This objective was addressed by exposing these rats to representative components of GCR combined with HLU and measuring perivascular cardiac fibrosis, gene variants, immune system dysregulation, cytokines, and calcium metabolism over a stopping point of 270–360 days. The whole genome was sequenced to identify gene variants known to be associated with clonal hematopoiesis. All studies were performed by investigators blinded to the treatment groups at the Medical College of Wisconsin (MCW), WI, USA. The number of subjects in each experimental group was based on our previous experience with similar measurements in similar studies. Cardiac perivascular fibrosis, the primary endpoint in tissue, and biomarkers of calcium metabolism, immune system activation, cytokines, and gene variants, conventional indicators for cardiac disease and kidney injury, the secondary endpoints, were selected prospectively. Stopping points for data collection were determined prospectively. Cardiac perivascular fibrosis was observed with a sample size of three hearts per group for sham irradiated and six hearts per group for irradiated rats based on previous studies using a similar experimental design and effect size [17]. This aspect of the experimental design allowed the conduct of additional studies on components of the immune system in heart to generate hypotheses for future larger studies [18,19]. To randomize animals in the experiment, a random number generator was used to ensure each animal received an equal chance at inclusion in any group to eliminate bias and was then assigned a unique ID. The identity of the animal in each experimental group was known to the investigator responsible for initiating and continuing with an intervention, for example, irradiation. The identity of the animal under study was not known to the investigator performing the experimental measurement or the data analysis. These investigators were not the same person. The start of the study was defined as the time rats began hind limb unloading. All data collected for primary and secondary endpoints were included in the study. Raw data are provided in supplementary data files.

### Experimental animals

Seventy-two male WAG/RijCmcr rats were bred at the MCW and were transferred at 8–9 months of age (n = 12/group) to BNL by commercial air courier with access to food and gel. This strain of rat was selected for study as exposure to three ion beams (protons, silicon ions, and iron ions) representative of GCR [2] results in cardiovascular disease in a time- and dose-dependent manner. This rat age corresponds to an age representative of the astronaut corps. Group sizes were based on a power analysis that indicated that for each dose/condition, data from 12 rats would detect the anticipated effect at a significance level of 0.05 with a power level of 0.80 for conventional biochemical indicators of cardiac and kidney injury [2]. The rats were acclimatized at BNL for 2 weeks before irradiation. The start of the study was defined as the time rats were hind limb unloaded, or sham treated. Animals were pair-housed and maintained on standard food (Teklad 8904 irradiated rodent diet, [20] and drinking water provided *ad libitum* throughout the experiments. The rats were provided with nesting material, maintained on a 12-hour light, 12-hour dark cycle at a temperature of 20 ± 1°C, and relative humidity 50–80%. Animals were monitored at least daily for signs of distress, including ruffled fur, lethargy, or reduced food intake. Animals were euthanized at the end of the study or when humane endpoints were reached. Humane endpoints for euthanasia were a decrease of 20% or more from baseline body weight, persistent hypothermia or a rapid decrease in core temperature for several consecutive days, an increase in respiratory rate, lethargy, recumbency, failure to reach food and water, ruffled fur, lack of grooming, or abnormal body posture. Once animals reached endpoint criteria they were euthanized immediately. Animals were euthanized by overdose of the anesthetic isoflurane followed by pneumothorax Animal studies were conducted in strict accordance with the US National Research Council's Guide for the Care and Use of Laboratory Animals, the US Public Health Service's Policy on Humane Care and Use of Laboratory Animals, NIH Guide for the Care and Use of Laboratory Animals, and by ARRIVE guidelines. The Animal Care and Use Committee at MCW (AUA4086) and BNL (482) approved all live animal experiments, and all efforts were made to minimize suffering and distress. Research staff, animal care personnel and veterinarians supervised the welfare of the animals. To minimize handling stress, animals were moved using cupping rather than tail picking. Research staff in the study had previous experience in animal care and handling of rats. Seventy two animals were used and 71 were euthanized. One control rat died during the third week of the study of unknown causes, determined from necropsy. The death was reported to the Institutional Animal Care and Use Committee and investigated. The remaining data for this group is based on n = 11. There were no clinical signs of infection or deaths in WAG/RijCmcr rats from HLU or from radiation during their stay at BNL or at MCW. All living subjects were followed throughout the full 270–360-day duration of the experiment. The composition of the experimental groups is shown in Table 1.

**Table 1:** Experimental groups.

Experimental group	Follow up (days)	Measurements made
1.5 Gy no HLU	30 - 270	Perivascular cardiac collagen content, serum calcium dynamics, immune cells in blood and tissues, serum cytokines, whole genome sequencing, serum cholesterol and triglycerides, blood urea nitrogen, total protein and albumin, body weight.
Sham irradiation no HLU	30 - 270	Perivascular cardiac collagen content, serum calcium dynamics, immune cells in blood and tissues, serum cytokines, whole genome sequencing, serum cholesterol and triglycerides, blood urea nitrogen, total protein and albumin, body weight.
1.5 Gy with HLU	30 - 270	Perivascular cardiac collagen content, serum calcium dynamics, immune cells in blood and tissues, serum cytokines, serum cholesterol and triglycerides, blood urea nitrogen, total protein and albumin, body weight.
Sham irradiation with HLU	30 - 270	Perivascular cardiac collagen content, serum calcium dynamics, immune cells in blood and tissues, serum cytokines, serum cholesterol and triglycerides, blood urea nitrogen, total protein and albumin, body weight.
0.75 Gy no HLU	360	Perivascular cardiac collagen content
Sham irradiation no HLU	360	Perivascular cardiac collagen content

### Irradiation conditions and dosimetry

High energy-charged particle beams were produced at the NSRL at BNL. The whole body of rats was exposed to a five-ion group given as 6 individual beams (simGCRsim) designed to simulate the composition of GCR behind spacecraft shielding (Table 2, [3]. The whole body of non-anesthetized rats was sham-irradiated or exposed to 0.75 Gy or 1.5 Gy of the simGCRsim delivered at high dose rates with rapid switching at the NSRL between May 7–8, 2019. Exposed animals were oriented in the beam line with their sides perpendicular to the beam to ensure uniform irradiation throughout the rat. The 1.5 Gy cohort included groups of simulated weightlessness using HLU or HLU combined with simGCRsim. A 60 x 60 cm, uniform irradiation field was used for these studies. The rats were positioned in the initial plateau region of the depth dose distribution in the field from the different ion beams provided by the NSRL facility. This produced a homogenous dose distribution of high energy charged particles within the rats [3]. The primary method of calibrating the dose delivered at NSRL was *via* an EGG calibration ion chamber (Far West Technology, Goleta, CA.). The Physics staff performed dosimetry at the NSRL [21].

**Table 2:** Five-ion mixed field normalized to 1500 mGy (=1.5 Gy).

Ion Species	Energy (MeV/n)	LET (keV/μm)	Dose (mGy)	Dose fraction	Delivery order
**Proton**	1000	0.2	525	0.35	1
^**28**^ **Si**	600	50.4	15	0.01	2
^**4**^ **He**	250	1.6	270	0.18	3
^**16**^ **O**	350	20.9	90	0.06	4
^**56**^ **Fe**	600	173.8	15	0.01	5
**Proton**	250	0.4	585	0.39	6

### Hind limb unloading

On Day 1, the rats were randomly grouped to serve as either full weight-bearing on all four limbs or HLU via tail suspension with body weight borne on the two front limbs. The HLU procedure for these same rats has previously been published [15,16,22]. Briefly, rats were lightly anesthetized with isoflurane (2.5–3.0%; 100% oxygen flow rate). Strips (1 cm wide x 30 cm long) of medical traction tape were placed laterally along the lateral sides of the tail (3M, St. Paul, MN, United States), originating approximately 1 cm from the base of the tail and then spanning 3/4ths the length of the tail. The tape was looped through a custom-built plastic ball bearing swivel then adhered to the opposing lateral side of the tail. A 1.56-in carabiner clip (Nite Ize, Boulder, CO, United States) was attached to the plastic swivel, and the other end of the carabiner was attached to a zipper hook that permitted sliding along a perlon cord (STAS group, BA, Eindhoven, Netherlands). The cord was attached to a support separator spreader pulley (Blue Hawk, Gilbert, AZ, United States). The pulley was able to slide the length of a 5/16-in diameter round, solid steel rod (Hillman, Cincinnati, OH, United States). The zipper lock was adjusted to lift the hind limbs off the floor with an angle of the thorax and abdomen 30^o^ to the floor. The rats maintained under full weight bearing (non-HLU) received isoflurane but were not tail suspended. All HLU and non-HLU rats were sub-grouped to receive a radiation dose (0.75 Gy or 1.5 Gy) or sham exposure (0 Gy); all animals were singly housed.

Wet rodent chow and gel packs were provided to ensure hydration. Corrugated cardboard bedding was supplied to permit burrowing for all rats. On Day 5, animals were transferred from HLU or non-HLU housing to another plexiglass box for radiation or sham-radiation exposure. HLU animals remained tail suspended during the entire transfer process by removing the ball bearing from the carabiner and moving the rat to a plastic plexiglass holder (9.5” x 4” x 7.5”), and plastic bead chains were attached to the swivel. These holders were placed into a rigid foam frame, allowing six rats (in six holders) to be placed into the beamline simultaneously. The custom frame permitted plastic bead chains to hang from a top crossbar. Each bead chain was looped through the hole in the swivel, and the free end was affixed to itself using a plastic zip tie. The bead chain was adjusted at the cross bar to ensure the rat’s body was 30^o^ relative to the plexiglass floor; the hind limbs continued to be lifted from the floor. The holder lid permitted the tail to extend through and to allow movement in the holder. Rats that did not receive HLU were placed into similar plexiglass boxes drilled with airholes and stacked into rigid foam frames but remained fully weight-bearing. Rats were then transported to the NSRL facility on each irradiation day for sham irradiation or radiation exposure. All methods were carried out per relevant guidelines and regulations. The HLU procedure was initiated 5 days before sham-irradiation or irradiation. The rats remained in the HLU condition 25 days after sham-irradiation or irradiation and were reloaded. The animals were group housed. One week following the end of the HLU period, the animals were shipped back from BNL to MCW by commercial air courier with access to food and gel.

### Histology

To evaluate tissue damage at 270 days or 360 days after irradiation, the entire heart (*n* = 3–6/group) was isolated from anesthetized irradiated and sham-irradiated (control) rats and fixed in 10% formalin (v/v) using our standard procedures as described elsewhere [23]. We consistently obtained transverse heart tissue sections from the middle of both ventricles. Before embedding, the heart was oriented between the base and the apex. Fixed tissue samples were embedded in paraffin with heart samples in the transverse plane. Sections 4 µm thick were cut from each block and stained with Masson’s-trichrome blue according to standard methods described elsewhere [23]. Ten sections from each heart were used for morphometric analysis as described elsewhere in a blinded fashion [23]. All selected images of the heart microvasculature were acquired using a Nikon E-55i microscope equipped with a Nikon DS-Fi1 camera. The collagen content was measured in the perivascular and wall space areas on calibrated images (25–35/rat/group) by planimetry and quantified using MetaMorph image analysis program version 7.7.0.0. (Molecular Devices, Downingtown, PA, USA). Planimetry determined the true area of the coronary vessel, thereby eliminating errors that may have been caused by vessel compression and collapse. Sections of both ventricles of the heart (n = 3–6/group) were analyzed in a blinded fashion. Randomly selected images of tissue were acquired using a Nikon Plan Fluor 403/0.75 objective lens mounted on a Nikon E-55i microscope fitted with a Nikon DS-Fi1 camera and saved as 24-bit RGB images in TIFF format. Perivascular fibrotic changes in 20–30 fields containing coronary vessels, 2–50 microns in diameter for each heart were quantified using MetaMorph image analysis program version 7.7.0.0. (Molecular Devices, Downington, PA). On calibrated images, the areas of both the vessel lumen and the perivascular space were measured by planimetry. Planimetry determined the true area of the coronary vessel, thereby eliminating errors that may have been caused by compression and collapse. The area of fibrosis, per luminal area, was then calculated for each coronary vessel accessed. The resultant data was then averaged per animal and then grouped by treatment for statistical analysis. The average microvasculature fibrosis was expressed as percentage collagen per lumen area for each image. The collagen content of the coronary vessels was defined as the total area stained blue with trichrome, expressed as a percentage of the lumenal area, and then averaged per animal and grouped by treatment for statistical analysis. Perivascular cardiac fibrosis was defined as an increase in collagen content in irradiated hearts above the average values for sham-irradiated animals [2,24,25].

### Indicators of risk for cardiac disease

Blood was withdrawn by venipuncture from the jugular vein at 30-day intervals beginning 30 days post-exposure and up to 270 days after whole body irradiation (1.5 Gy) with HLU, whole body irradiation (1.5 Gy) no HLU, sham-irradiation with HLU and sham-irradiation no HLU. Collected blood was allowed to clot at room temperature, centrifuged (1,000g for 10 min at 4^o^C) to separate the serum from blood cells, and stored at −80°C until analysis. The serum was then analyzed for total cholesterol and triglycerides (Roche Cobas 8000 modular analyzer series, c701 module, Wisconsin Diagnostic Laboratories, Milwaukee, WI, a CLIA-certified laboratory).

### Kidney injury, metabolic, calcium dynamics, and cytokine biomarkers

Blood was withdrawn by venipuncture from the jugular vein at 30-day intervals beginning 30 days post-exposure and up to 270 days after whole body irradiation and HLU, whole body irradiation no HLU, sham-irradiation with HLU and sham-irradiation no HLU. Serum was analyzed for blood urea nitrogen (BUN), total protein, albumin, and calcium (Roche Cobas 8000 modular analyzer series, c701 module, Wisconsin Diagnostic Laboratories, Milwaukee, WI). Serum inorganic phosphorus was measured by UV spectrophotometer (Pointe Scientific Inorganic Phosphorus Reagent Set (UV) P7516-500, Canton, MI 48188 USA). Serum concentrations of calcium-regulating hormones were measured by ELISA validated for rat serum as follows: 25-hydroxyvitamin D3 (IDS 25-Hydroxy Vitamin D^S^ AC-57SF1, Boldon, Tyne & Wear, NE35 9PD UK), intact parathyroid hormone (Quidel MicroVue Rat Intact PTH 60–2500, Athens, OH 45701 USA), osteocalcin (Immunotopics Rat Osteocalcin 60–1505, San Clemente, CA 92673, USA), and fibroblast growth factor 23 (Immunotopics Mouse/Rat FGF-23 Intact 60–6800, San Clemente, CA 92673, USA). Serum samples were analyzed for circulating cytokines using a validated rat 27-plex Discovery Array (Eve Technologies Co, Calgary, AB, Canada).

### Immune cell isolation and flow cytometry

Immune cell isolation and flow cytometry were performed as previously described [26]. Peripheral blood mononuclear cells were isolated from heparinized whole blood over Histopaque-1083 (Cat# 10831, Sigma-Aldrich). Approximately 1X10^6^ cells were stained with fluorochrome-bound antibody cocktail containing anti-CD45 PE-Cy7 (BioLegend, catalog no. 202214, clone OX-1, 0.062 µg), anti-CD3 PerCP eFluor710 (eBioscience, catalog no. 46-0030-82, clone G4.18, 0.062 µg), anti-CD8a FITC (BioLegend, catalog no. 201703, clone OX-8, 0.125 µg), anti-CD4 APC-Cy7 (BioLegend, catalog no. 201518, clone W3/25, 0.062 µg), anti-CD11b/c Alexa eFluor 660 (eBioscience, catalog no. 50-0110-82, clone OX-42, 0.062 µg), and anti-CD45R PE (BD Bioscience, catalog no. 554881, clone HIS24, 0.125 µg) in 100 µl of wash buffer (PBS [x1] without Ca2+ and Mg2 + , 2% FBS, and 2 mM EDTA). Excess antibody was washed, and cells were resuspended in 300 µl of wash buffer. DAPI was added to quantify dead cells 10 min before samples were run on the flow cytometer. Flow cytometry was performed on an LSR II flow cytometer (BD Biosciences, San Jose, CA), and data analysis was performed with FlowJo Software (FlowJo, Ashland, OR).

### Immunohistochemistry

Heart tissue and spleen paraffin-embedded sections were stained immunohistochemically using a DAKO Autostainer Plus automated staining platform (Agilent/DAKO, Santa Clara, CA). The antibodies investigated included T cells with DAKO Rabbit Polyclonal CD3^+^ (A0452, 1:100), B cells with Cell Signaling Rabbit Monoclonal CD20^+^ (48750, 1:600), natural killer cells with Cell Marque mouse monoclonal CD56^+^ (156R-94, 1:200), and macrophages with EMB Millipore mouse monoclonal (CD68^+^ (MAB1435, 1:100). Pretreatment was performed with citrate buffer for CD3^+^, CD20^+^, CD56^+^ and CD68^+^, using EDTA retrieval. The standard Labeled Streptavidin Biotin approach was applied for detection with all antibodies. Following pretreatment and blocking steps, primary antibodies were incubated for 1 hour at room temperature. Biotinylated secondary antibodies were incubated for 30 minutes at room temperature (anti-mouse Jackson Immuno 715-066-151 and anti-rabbit Jackson Immuno 715-066-152) followed by a 15-minute incubation with streptavidin-HRP (DAKO P039701-2). Antibody visualization was achieved with the DAB+ application (DAKO DAB + K346811-2). All slides were counterstained with Modified Mayer’s Hematoxylin (DAKO S3309330-2) blued with 0.1% ammonium water and mounted with a synthetic mounting media. The omission of the use of primary antibodies served as a negative control. Antibodies were tested and validated for immunohistochemistry (IHC) by the Histology Core Facility at Children’s Hospital of Wisconsin [27]. The IHC images were digitally recorded using a high-resolution, whole slide scanner (NanoZoomer HT 2.0, Hamamatsu, Japan) at 40x magnification, and the data were reviewed using NDPview (version 2.7.43, Hamamatsu, Japan) for virtual image exploration. The scanned images were imported using Image Analysis Software (Visiopharm, Denmark) and analyzed at 20x magnification to quantify the DAB expressions. The software was trained to capture DAB-positive areas in the spleen and heart using a preset threshold and the linear Bayesian classification [28]. The processed images were pseudo-color-coded for DAB-positive areas and tissue areas for each spleen section, and the data was expressed as a percentage of the total tissue area. The IHC images were recorded and analyzed at the Children’s Research Institute Imaging Core at the MCW. The antibodies used for the immune cell staining of hearts were not of high enough quality to be included in the manuscript.

### Whole genome sequencing of nucleated cells from peripheral blood

We sequenced the whole genome of circulating, nucleated cell populations in the WAG/RijCmcr rat and used bioinformatics to detect alterations in samples from rats previously exposed to 1.5 Gy of simGCRsim, with no HLU or in sham-irradiated rats also with no HLU. The samples were collected at 240 days following the start of the study. With the rat genome covering approximately 2.75 billion bases, we sequenced 6 samples (3 exposed, 3 sham-exposed) with paired-end 115 bp reads on an S2 NovaSeq flowcell that provided 1.4 billion reads for each sample, for an approximate coverage of 60x across the genome. Using the outcomes of this limited study, we based our initial analysis on genes known to be involved in leukemogenesis in humans and in mice. This bioinformatic-based panel focused on 74 genes previously associated with CHIP [14,29]. We determined the observed variant allele numbers in these 74 genes. This data was generated using the same analytical approach as a certified clinical genomics laboratory uses for patients. Thus, this data demonstrates our abilities and the feasibility to conduct the proposed studies to detect genomic changes associated with CHIP. We calculated the VAF of each genetic variation using the ratio of sequencing reads that detect the alternate allele compared to total read depth at the same site. Geneset and pathway term enrichment and depletion used the Gene Ontology (GO, [30,31] and KEGG [32] resources and hypergeometric statistics, as implemented in a platform from the Ge lab [33].

### Statistical analysis

All endpoint values apart from the gene variant analysis were expressed as the mean ± standard deviation (SD) or standard error of the mean (SEM). The statistical strategy was selected based on the structure of each dataset and the experimental design. For continuous longitudinal endpoints involving repeated measurements within the same animals, linear mixed-effects modeling was used to appropriately account for within-subject correlation, time-dependent effects, and incomplete observations where applicable. For single-timepoint endpoints, analyses were performed using the statistical approach most appropriate to the specific design and distributional characteristics of the data. This study is focused on the biological effects of a combined stressor exposure to simGCRsim and HLU to directly evaluate whether the effects of combined exposure differ from the expected additive effects of the individual stressors. p-values from multiple comparisons were adjusted using the Benjamini–Hochberg false discovery rate correction to control for multiplicity while maintaining sensitivity appropriate for a multidimensional biological dataset [34] with a q-value cutoff of 0.1 (S1 Table). For secondary, non-longitudinal endpoints where mixed models were mathematically inappropriate, two-group comparisons were performed using the t-test. Statistical significance was defined at an adjusted p value of < 0.05. Perivascular cardiac collagen and calcium dynamics data were analyzed using Sigma Plot 11 software. All data were examined for normality using Shapiro-Wilk test [35]. Perivascular cardiac collagen data were analyzed using the t-test. Testing for statistical significance for the calcium dynamics data was performed using repeated measures analysis of variance (ANOVA) with all pairwise multiple comparison procedures (Holm-Sidak method) or Kruskal-Wallis One Way ANOVA on ranks when normality test failed. Individual ‘p’ values at specific time points in the longitudinal studies are reported in the figures.

## Results

There were six experimental groups (Table 1). Body weight increased in the simGCRsim with HLU group before the start of the study compared with other groups. This was attributed to randomization of rats. Body weight increased during the study and was unchanged in the simGCRsim with the HLU group at the end of the study compared with other groups (S1 Fig).

### SimGCRsim plus HLU causes perivascular cardiac fibrosis

Staining of heart slices with Trichrome revealed peri-arterial fibrosis and irregular collagen deposition around the penetrating coronary vessels from rats 270 days after 1.5 Gy no HLU and 360 days after 0.75 Gy no HLU (Fig 1). The vessel lumen was blocked because of myointimal proliferation. Hearts from age-matched, sham-irradiated rats no HLU had symmetrical penetrating vessels with less collagen and fibrosis. Cardiomyocytes from irradiated and HLU rats remained normal in appearance (Fig 1). Exposure of rats to 0.75 Gy or 1.5 Gy no HLU resulted in a specific observable effect manifested as a 2-fold increase in perivascular cardiac collagen deposition after 360 days and 270 days respectively, compared with sham-irradiated no HLU (Fig 2). The incidence of cardiac fibrosis in rats exposed to a total dose of either 0.75 Gy or 1.5 Gy no HLU was 83% and 100% respectively. Whole body exposure to 1.5 Gy no HLU increased cardiac perivascular collagen content compared with sham-irradiated no HLU evaluated after 270 days (p = 0.024). Exposure of HLU rats to 1.5 Gy resulted in an increase in cardiac perivascular collagen content compared with sham irradiated rats with no HLU evaluated at 270 days (p = 0.008). However, adding HLU to a 1.5 Gy dose of simGCRsim alone resulted in cardiac perivascular fibrosis but did not change the degree of fibrosis beyond the effect of 1.5 Gy alone at the same timepoint. The two groups could not be distinguished. HLU alone did not cause cardiac perivascular fibrosis (p = 0.223). In the follow-on study, exposure to 0.75 Gy of simGCRsim, a dose relevant to upcoming human missions to Mars, no HLU resulted in cardiac perivascular fibrosis after 360 days (p = 0.031).

**Fig 1:**
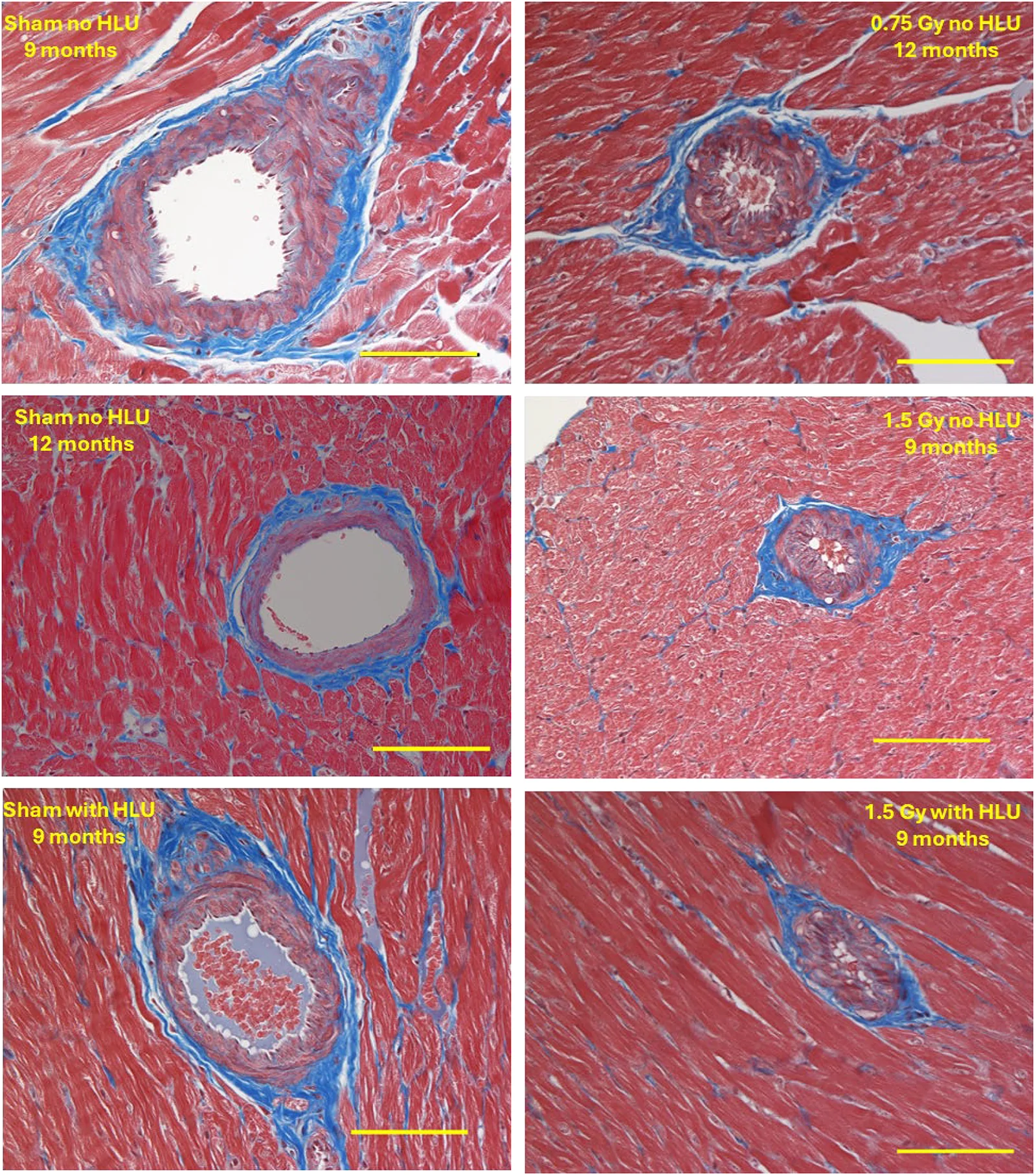
Structural changes in the heart 270 days and 360 days after simGCRsim and HLU or sham irradiation no HLU. Rats were irradiated with 0.75 Gy or 1.5 Gy simGCRsim with no HLU, 1.5 Gy with HLU and compared with no HLU or sham-irradiated groups. Rats were 8-9 months of age at the start of the study and 17-18 or 20-21 months at the end. Heart sections were stained with Trichrome. The horizontal bar represents 100 microns. Images are representative of data from 3-6 animals per group. *n* = 3-6 animals per group.

**Fig 2:**
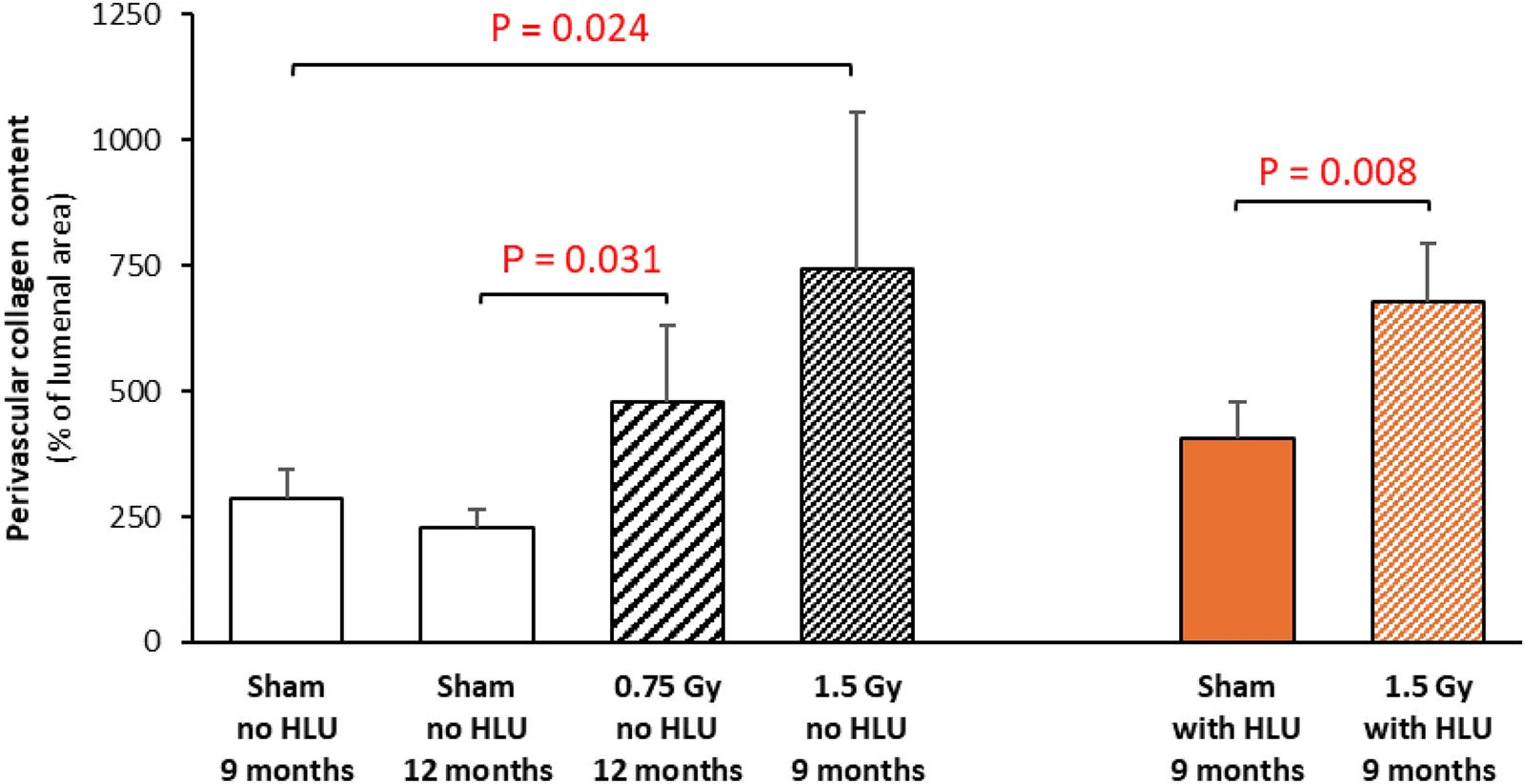
Perivascular cardiac collagen content in hearts 270 days and 360 days after simGCRsim and HLU or sham irradiation no HLU. Rats were irradiated with 0.75 Gy or 1.5 Gy simGCRsim with no HLU, 1.5 Gy with HLU and compared with no HLU or sham-irradiated groups. Rats were 8-9 months of age at the start of the study and 17-18 or 20-21 months at the end. The perivascular collagen content was defined as the total area of the media plus adventitia stained blue with trichrome, expressed as a percentage of the lumenal area. Data are mean ± SD, n = 3-6 /group. Statistical analysis was conducted using a t-test.

### HLU and simGCRsim disrupt calcium dynamics

To determine the impact of reloading after HLU and discontinuation of simGCRsim on calcium homeostasis, peripheral blood was collected over the 270-day study period to analyze total calcium levels. Sham irradiation with HLU alone decreased serum calcium at 60, 90, 120 and 210days compared with age-matched sham-irradiated no HLU rats (Fig 3A). Calcium levels increased at 150 days. 1.5 Gy simGCRsim no HLU decreased serum calcium at 90, 120, 210, and 240 days compared with sham-irradiated no HLU rats (Fig 3B). 1.5 Gy simGCRsim with HLU decreased calcium levels at 120, 210, and 270 days compared to sham-irradiation no HLU and increased at 90 days (Fig 3C). Albumin levels were unchanged by simGCRsim and HLU over the 270-day study period, thus no corrections to total calcium values were needed (Suppl. S2 Fig). To demonstrate a role in endocrine regulation of calcium metabolism, serum concentrations of 25-hydroxyvitamin D_3_, parathyroid hormone, osteocalcin, fibroblast growth factor 23, and inorganic phosphorus were measured over the 270-day study period. Sham irradiation with HLU decreased serum 25-hydroxyvitamin D_3_ at 210 days compared with age-matched sham-irradiated no HLU rats. Serum 25-hydroxyvitamin D_3_ concentrations were increased at 60 and 180 days (Fig 3D). 1.5 Gy simGCRsim no HLU did not affect serum 25-hydroxyvitamin D_3_ levels (Fig 3E). 1.5 Gy simGCRsim with HLU decreased serum 25-hydroxyvitamin D_3_ levels at 150 and 270 days. 25-hydroxyvitamin D_3_ levels increased at 180 days. (Fig 3F). However, the observed changes were relatively modest in magnitude. Sham irradiation with HLU, 1.5 Gy simGCRsim no HLU, and 1.5 Gy simGCRsim with HLU did not affect serum parathyroid hormone, osteocalcin, fibroblast growth factor 23, and inorganic phosphorus over the 270-day study period compared with age-matched controls (Suppl. S3 Fig).

**Fig 3:**
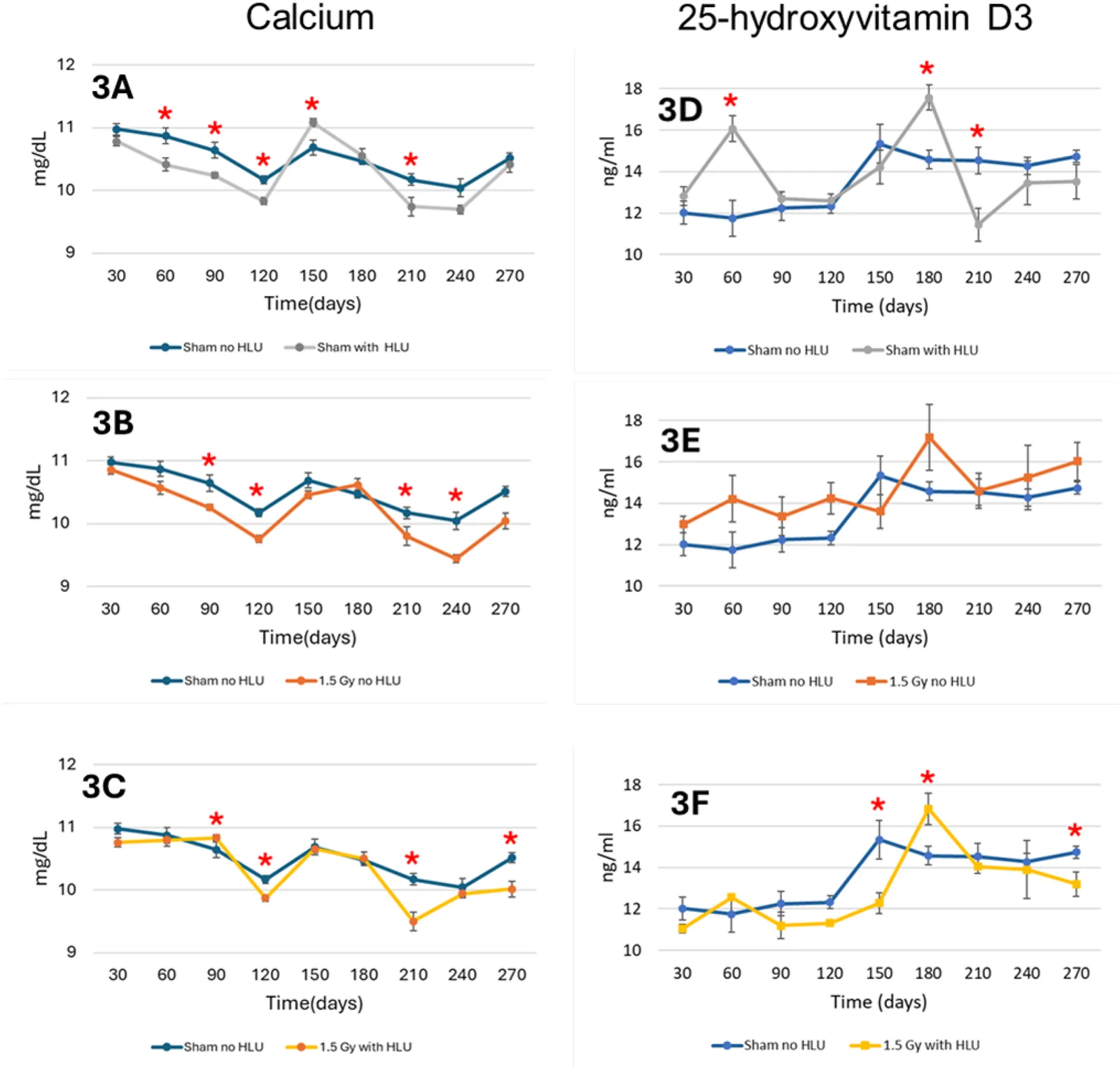
Biomarkers of calcium metabolism. Comparison of group differences in serum calcium and 25-hydroxyvitamin D_3_ levels affected by HLU and simGCRsim over time. Data represents the mean ± SE, *n* = 3 - 6 rats per group. * = p < 0.05 vs. age-matched control using t-test at individual time points.

### simGCRsim abolishes activation of the immune system in peripheral blood by HLU

The lymphoid and myeloid immune cell levels in peripheral blood were measured 240 days after the start of the study. Exposure of rats to 1.5 Gy simGCRsim alone, no HLU did not change the levels of total leukocytes (CD45^+^), T lymphocytes (CD3^+^), helper T lymphocytes (CD3^+^/CD4^+^), cytotoxic T lymphocytes (CD3^+^/CD8^+^), B lymphocytes (CD45R^+^), and myeloid cells (CD11bc^+^) in peripheral blood compared with sham-irradiated rats no HLU (Fig 4). Exposure to sham-irradiation with HLU increased the numbers of circulating CD45^+^, CD3^+^, CD3^+^/CD4^+^, CD3^+^/CD8^+^ and CD45R^+^ lymphocytes (p < 0.05) but not the numbers of CD11bc^+^ myeloid cells compared with sham-irradiation no HLU (Fig 4). Exposure to 1.5 Gy with HLU did not change the levels of CD45^+^, CD3^+^, CD3^+^/CD4^+^, CD3 + /CD8^+^, CD11bc^+^ and CD45R^+^ levels compared with the sham irradiated no HLU group (Fig 4).

**Fig 4:**
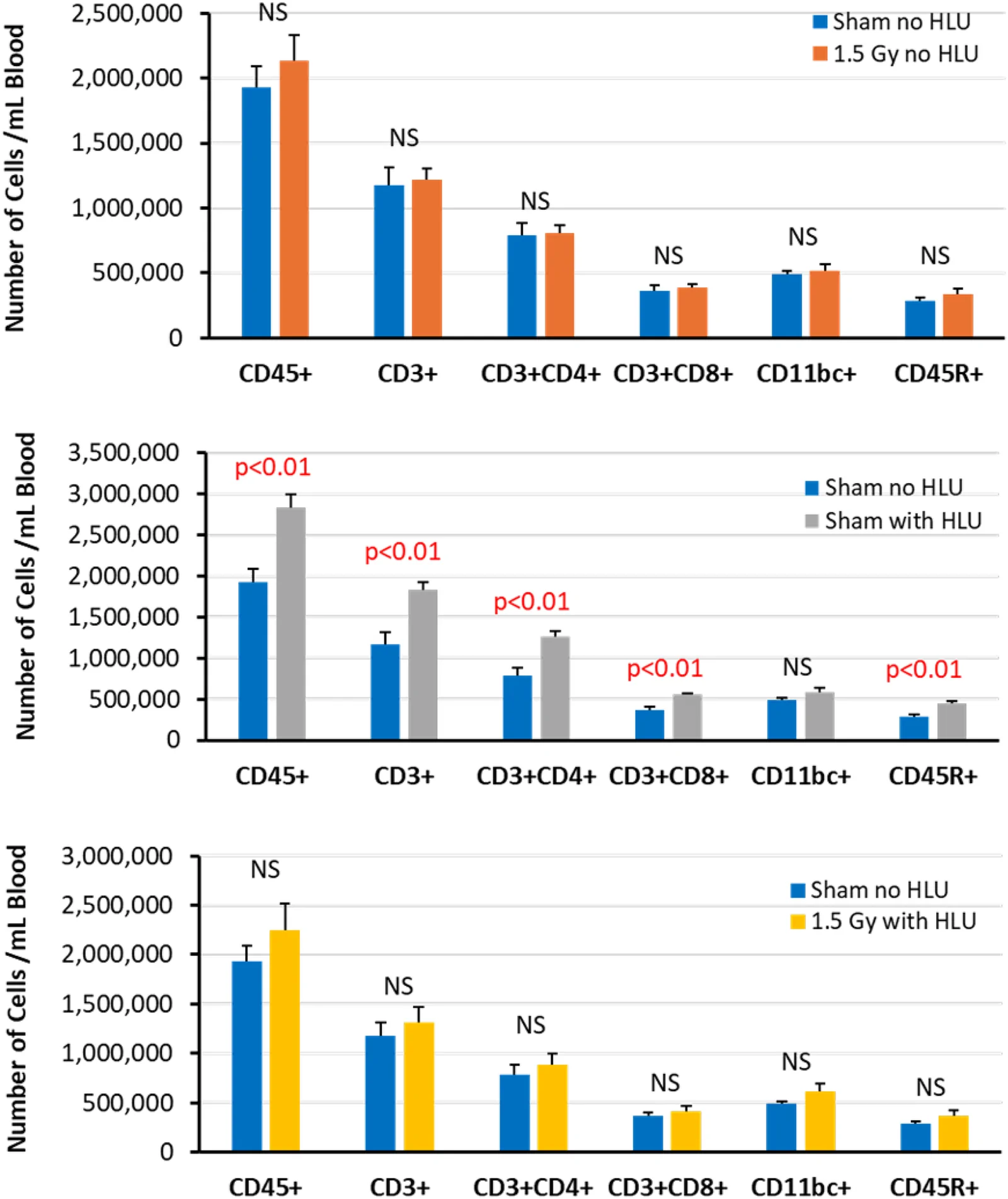
Measurement of numbers of discrete components of the immune system in peripheral blood. Immune cell numbers of total leukocytes (CD45^+^), T lymphocytes (CD3^+^), helper T lymphocytes (CD3^+^/CD4^+^), cytotoxic T lymphocytes (CD3^+^/CD8^+^), myeloid cells (CD11bc^+^) and B lymphocytes (CD45R^+^) in circulation 240 days after whole body exposure to simGCRsim, HLU, and simGCRsim plus HLU. NS = not significant. Data are mean ± SD, *n* = 6 animals per group. Statistical analysis was conducted using a t-test.

### HLU and simGCRsim deplete Natural killer cells in the spleen

To measure the levels of immune cells in the spleen in response to whole-body exposure to simGCRsim and HLU, immunohistochemistry (IHC) was performed on sections of spleen tissue from rats 270 days after the start of the study. In sham irradiated no HLU rats, there were areas positive for biomarkers of T cells (CD3^+^), B cells (CD20^+^), natural killer cells (CD56^+^), and macrophages (CD68^+^) in the spleen. Exposure to sham-irradiation with HLU decreased areas positive for biomarkers of CD56^+^ compared with sham-irradiation no HLU (p = 0.007,Fig 5). Exposure to 1.5 Gy simGCRsim with HLU decreased areas positive for biomarkers of CD56^+^ compared with 1.5 Gy simGCRsim no HLU group (p = 0.009,Fig 5). There were no changes in areas positive for biomarkers of CD3^+^, CD20^+^ and CD68^+^ in spleen from rats exposed to simGCRsim and/or HLU (Suppl. S4 Fig). These findings suggest that exposure to spaceflight stressors like HLU and simGCRsim can preferentially deplete Natural Killer cells in lymphoid organs like the spleen, while sparing other immune cell types like T cells, B cells, and macrophages.

**Fig 5:**
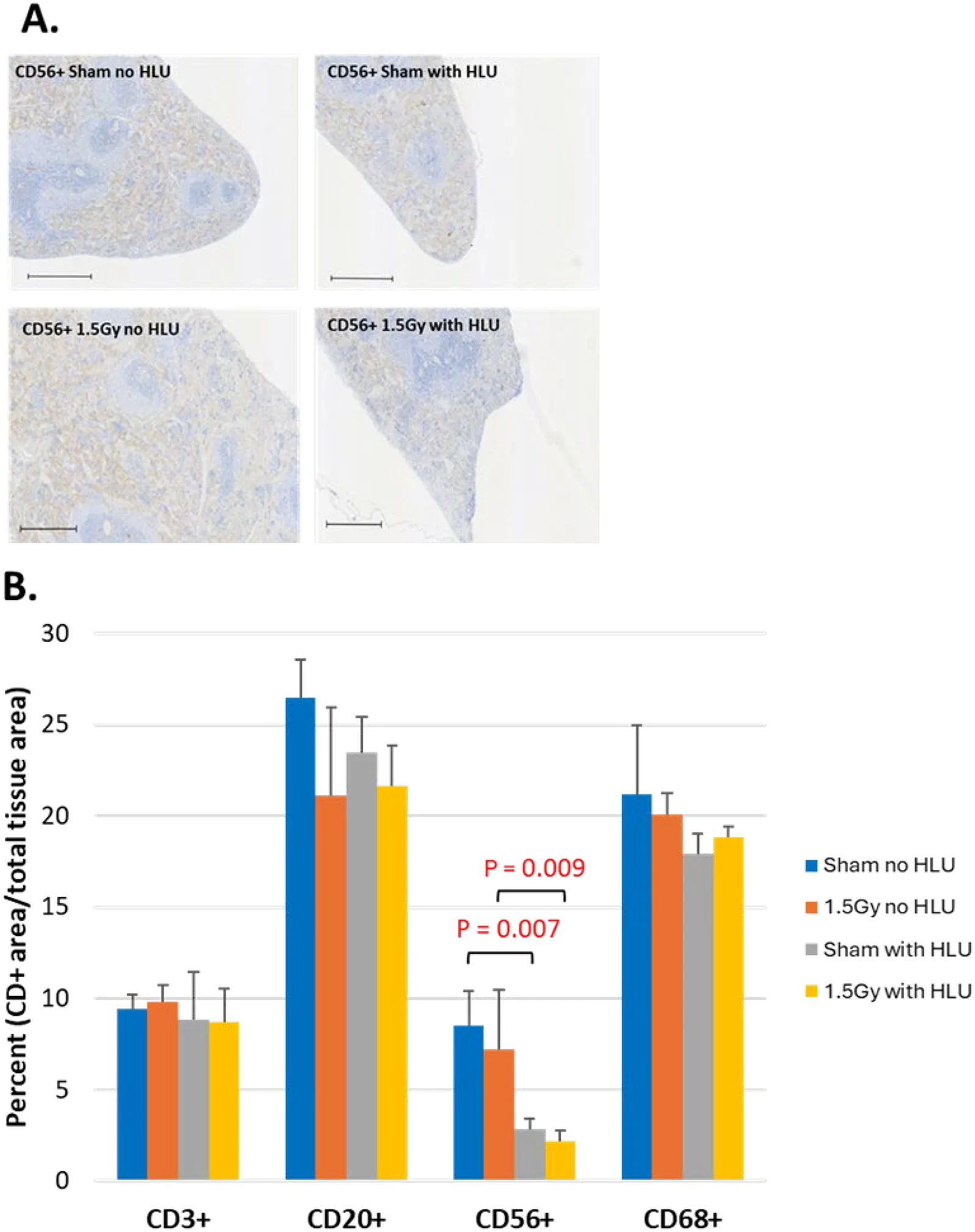
Depletion of components of the immune system in spleen. A. Areas positive for biomarkers of natural killer cells (CD56^+^) in spleen 270 days after sham irradiation no HLU, sham irradiation with HLU, whole body exposure to simGCRsim no HLU, and simGCRsim with HLU. The horizontal bar represents 500 microns. B. The CD^+^ content of the spleen was defined as the area of CD^+^ positive staining cells, expressed as a percentage of the total tissue area. Data are mean ± SD, n = 6 animals per group. Statistical analysis was conducted using a t-test.

### simGCRsim abolishes decreased cytokine production by HLU

We analyzed blood from rats 30–270 days after irradiation with 0.75 or 1.5 Gy simGCRsim and age-matched, sham-irradiated controls with and without HLU. A multiplex cytokine array was used to identify 27 signaling molecules connected to mechanisms underlying disease. All 27 cytokines could be consistently quantified.

Over the 30–270 day study period, sham-irradiation with HLU decreased levels of 20/27 (74%) of the cytokines measured compared with sham-irradiation no HLU: EGF, Eotaxin, Fractalkine, GM-CSF, GRO-KC, IFN gamma, IL-1 alpha, IL-1 beta, IL-2, IL-4, IL-6, IL-10, IL-12p70, IL-18, IP-10, Leptin, MIP-1 alpha, MIP-2, VEGF and RANTES (Fig 6). In contrast, neither 0.75 Gy simGCRsim alone nor 1.5 Gy simGCRsim alone affected cytokine release compared with sham-irradiation no HLU (S1 Table). The combination of 1.5 Gy simGCRsim with HLU did not affect cytokine release compared with sham-irradiation no HLU (S1 Table), suggesting that the radiation exposure inhibited changes elicited in cytokine profiles by HLU alone.

**Fig 6:**
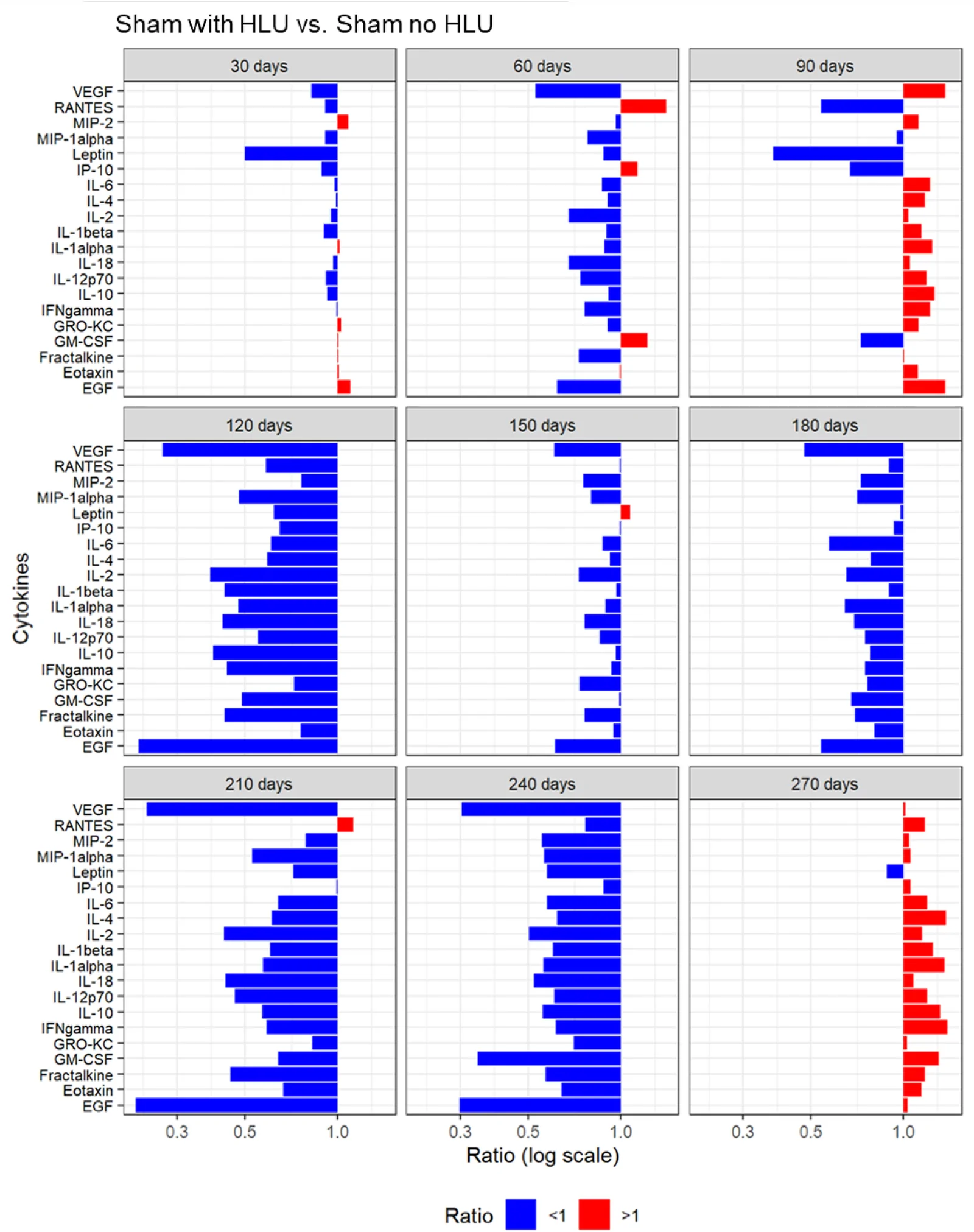
Comparative changes in cytokines present in the circulation 30 – 270 days after sham-irradiation with HLU compared with sham-irradiation no HLU. Ratios <1 show a relative decrease in cytokines for sham irradiation with HLU vs. sham irradiation no HLU. Ratios >1 show a relative increase in cytokines for HLU vs no HLU. *n* = 10 rats per group. A mixed effects model with random intercept was fitted to the log-transformed the value of each cytokine as the outcome variable. Fixed effects for sampling time and study group were included. Pairwise comparisons between the groups were performed, with no adjustment for multiple testing. Benjamini & Hochberg method (“fdr”) is used for p value adjustment between cytokines.

### Identification of Genomic areas with evidence of radiation-induced changes

We used next-generation sequencing on nucleated peripheral blood samples to detect gene variants in circulating cell populations in rats previously exposed to 1.5 Gy simGCRsim with no HLU compared with sham-irradiation with no HLU. Samples were taken on day 240 of the study. With the rat genome covering approximately 2.75 billion bases, we sequenced 6 samples (3 exposed to simGCRsim, 3 sham-exposed) with end paired 115 bp reads on an S2 NovaSeq flowcell that provided 1.4 billion reads for each sample, yielding 60x average coverage across the genome. Variant analyses considered insertions, deletions and single nucleotide polymorphisms. When considering the high-impact variants, we observed many gene variants overlapped among the sham and simGCRsim exposed rats (7224 variants), with unique variants also discovered for each condition, sham (964 variants) and simGCRsim (874 variants,Fig 7A). Further, we selected 74 genes previously associated with CHIP development in humans and mice [14,29] and determined the number of variants found in these 74 genes (Fig 7B). Variants were observed in 30 of 74 (41%) CHIP development genes examined with only seven gene variants identified uniquely in the simGCRsim condition (Fig 7B, green bars): *Smc3, Suz12, Brcc3, Ep300, Cblb, Idh2* and *Usaf2*. The 30 CHIP-associated genes were affected by 48 individual gene variants. We analyzed variant allele frequency (VAF) and additional metrics, which are further detailed in S2 Table. The variants in *Brcc3, Ep300, Cblb,* and *Usaf2* occurred in a range inconsistent with germline variation (VAF (%) <0.250), supporting somatic origin. To investigate interactions and relationships among the CHIP development genes altered, we performed mapping to the rat genome deposited in the Rat Genome Database [36]. Using the algorithm described by Ge *et al.* [33], genes were linked to biological pathways and assessed for statistical significance of the observations. Comparison of all CHIP variants (S3 Table) across experimental conditions unexpectedly yielded a set of variants that allowed us to predict potential damaging effects of GCR exposure. Notably, ontological analyses shown below (Table 3), using statistical methods of mapping to GO Cellular Component pathways, suggested that together, the CHIP-associated variants are involved in a continuum of signaling to transcriptional regulation (nuclear, chromatin, and chromosomal proteins). Notably, many of these proteins are involved in enhancer regulation in 3D, creating an open state in these nuclear regions *in vivo*, which may predispose to subsequent damage by radiation. Indeed, pathway reconstructions using knowledge-based algorithms demonstrate that many of the products of these genes interact in networks. Interestingly, we found that genetic variation in CHIP-associated genes between animals was the dominant signal, making the number of irradiation-associated variants like those in the sham group. This finding emphasizes the need for individualized studies that explicitly account for per-person or per-animal baseline genomics.

**Table 3:** Ontological analysis of CHIP gene variants.

Pathways	Fold Enrichment	Enrichment FDR	nGenes	Pathway Genes
Cohesin complex	247.5	1.30E-05	3	10
Nuclear protein-containing complex	7.3	1.30E-05	11	1239
Nucleoplasm	4	8.00E-05	14	2915
Nuclear matrix	39.3	1.00E-04	4	84
Mitotic spindle pole	66.9	2.30E-04	3	37
U2AF complex	275	3.30E-04	2	6
Nuclear meiotic cohesin complex	206.2	3.30E-04	2	8
Spindle pole	21.9	3.30E-04	4	151
Chromosome	5.9	3.30E-04	8	1126
Catalytic complex	5	3.30E-04	9	1485
Intracellular organelle lumen	3.1	3.30E-04	14	3740
Chromatin	7.6	8.00E-04	6	650
Transferase complex	6.2	2.40E-03	6	803
Sex chromosome	47.1	5.20E-03	2	35

**Fig 7:**
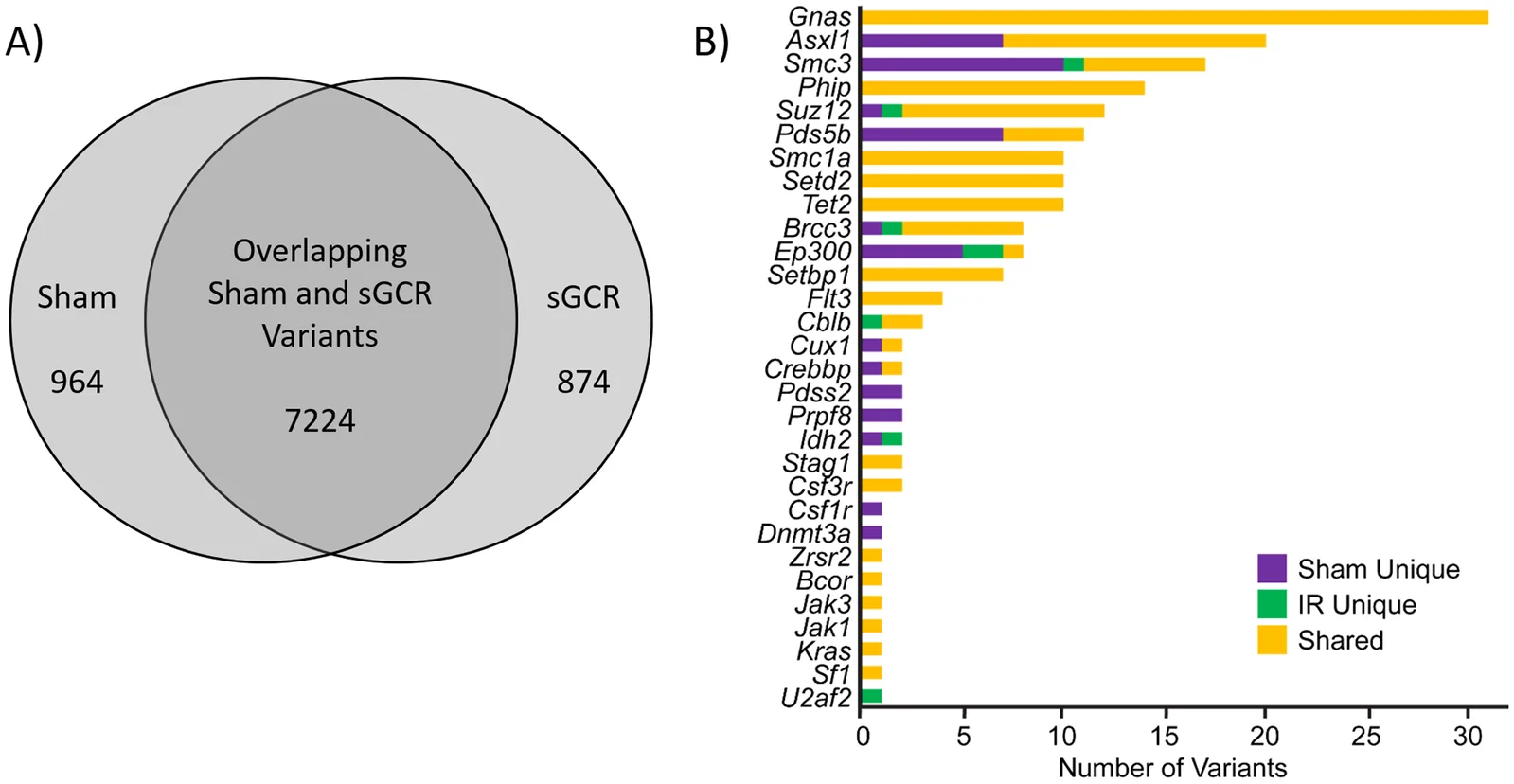
Analysis of gene variants identified in whole genome sequencing. A) Overlap and unique number of gene variants with high impact found in sham-irradiated and simGCRsim (1.5 Gy) rats. B) Gene variants with an impact associated with CHIP. *n* = 3 rats per group.

Shifting the focus to all high impact variants unique to the simGCRsim condition, defined as those that disrupt the coding frame, statistical methods identified three regions on chromosomes 1, 4A, and 4B where variants are enriched (FDR 0.05) when scanning the genome using 4MB windows and indicating areas of severe genome alteration. The cluster on chromosome 1 contains olfactory genes (*olr36* and *Olr37*) and *THAP12*, predisposes to Ataxia-telangiectasia-like disorder 2 [37,38]. The chromosome 4A cluster includes genes essential for both epigenomic regulation of enhancer regions in the genome (*KMT2e*), where alterations in humans predisposes to O'Donnell-Luria-Rodan syndrome, as well as being a critical regulator of the Alzheimer predisposition genes (*GSAP*, g-secretase activating protein). Both impact neurodevelopmental and neurodegenerative diseases (Alzheimer’s) with inflammatory components. Furthermore, the chromosome 4 distal cluster contains information relevant to inflammation (*Klrc2, Klra2, lys49s5,* and its Ly49si1like 1 receptor) and DNA repair (*Nutd5*). These analyses indicate e that simGCRsim affects the genome in a manner that could increase the likelihood of developing additional conditions of high medical relevance However, the absence of paired samples obtained pre- and post- exposure for individual animals for the two groups (1.5 Gy, no HLU vs. sham-IR, no HLU) and preferably longitudinal samples from individual animals over the full time course would permit detecting any effect of baseline variations in individuals and aging-associated changes in each group.

### simGCRsim and HLU minimally affect conventional indicators for cardiac disease

Blood total cholesterol, a risk factor for human cardiac disease, was unchanged in rats irradiated with 1.5 Gy of simGCRsim no HLU compared with sham-irradiated rats with no HLU (S5 Fig). Total cholesterol was also unchanged in rats sham irradiated with HLU compared with sham irradiated no HLU rats (S6 Fig). Total cholesterol was unchanged in rats irradiated with 1.5 Gy simGCRsim with HLU compared with sham-irradiated rats with normal weight bearing (S7 Fig). Triglycerides, a risk factor for cardiac disease, were essentially unchanged by simGCRsim and HLU compared with age-matched sham-irradiated and no HLU controls (S5–S7 Fig). With the extent of multiple comparisons made we cannot exclude that a few with a p < 0.05 were false positives.

### simGCRsim and HLU minimally affect conventional indicators for kidney injury

Blood urea nitrogen (BUN), an index of human kidney injury, was unchanged in rats irradiated with 1.5 Gy no HLU compared with sham-irradiated no HLU rats up to 270 days (S5 Fig). BUN was unchanged in sham-irradiated rats with no HLU compared with sham-irradiated rats with HLU (S6 Fig). BUN was elevated at 90 days in rats irradiated with 1.5 Gy with HLU compared with sham-irradiated rats with normal weight bearing (S7 Fig). Total protein levels were essentially unchanged by simGCRsim and HLU compared with age-matched, sham-irradiated and no HLU controls (S5–S7Fig).

## Discussion

During exploration missions extending beyond low earth orbit, astronauts will be continuously exposed to low levels of GCR as well as weightlessness and the stress of space operations and potential risks. Prior to undertaking such missions, we need to understand as much as possible in the time available about the impact of these spaceflight stressors on genetic, molecular and biochemical mechanisms in biological systems both during the mission as well as following the return to Earth. We utilized a ground-based animal model exposed to two spaceflight stressors – hindlimb suspension as a simulation of weightlessness (HLU) and radiation exposure simulating a 3-year deep space mission (0.75 Gy of simGCRsim) to determine increased risk for cardiovascular disease, disruption of the immune system, cytokines, gene variants, and changes in calcium homeostasis. A higher simGCRsim dose was included to benchmark the current study against our previous work [2]. Delivery and then discontinuation of simGCRsim exposures resulted in cardiac perivascular fibrosis, the primary endpoint. Exposure to 1.5 Gy simGCRsim no HLU caused marked genomic alterations in circulating cell populations and identified mutations, frameshifts, and deletions in genes, a secondary endpoint, that contribute to human disease. Reloading after HLU disrupted the immune system and cytokine production, secondary end points. These findings indicate a persistent health effect of each spaceflight stressor on multiple systems.

Whole-body exposure of rats to a five-ion six-beam simGCRsim suite of beams delivered in close succession, designed by NASA to simulate the composition of GCR behind spacecraft shielding, resulted in cardiac perivascular fibrosis. The simGCRsim findings confirm the same extent of cardiac perivascular fibrosis, measured as increased collagen content, caused by exposure to 1.5 Gy from three ion beams representative of GCR in the same rat strain and over the same study duration [2]. Here, a longer follow-up period after exposure to 0.75 Gy of simGCRsim, revealed cardiac perivascular fibrosis after 360 days. While exposure to 0.75 Gy from a group of 3 ion beams delivered in close succession in our earlier study led to increased perivascular collagen levels after 270 days, those results did not reach statistical significance in the more limited observation period [2]. Thus, the more extended follow-up period used here revealed detrimental cardiovascular outcomes at lower doses, demonstrating a lower apparent threshold for this potentially critical health outcome. Because radiation injury is inherently dynamic, evaluating dose without accounting for time fails to capture how biological systems express latent damage. Using our radiation model, where dose rate is intentionally kept constant, we demonstrate the bivariate relationship between absorbed radiation dose and post-exposure follow up time plays a critical role in the presentation of cardiac perivascular fibrosis. The present study confirms our previous findings of perivascular cardiac fibrosis 270 days after a dose of 1.5 Gy [2]. We now show exposure to a lower dose of 0.75 Gy with the follow up period extended to 360 days results in perivascular cardiac fibrosis. Thus, radiation dose and post-exposure follow up time determine the severity and progression of cardiac injury. Our findings highlight a critical challenge in space radiobiology that GCR induces complex, time-dependent damage that traditional, static dose calculations fail to properly account for. Reloading rats after HLU decreased levels of immune cells, cytokines, and calcium in peripheral blood. Astronauts who complete long-duration exploratory missions face potential chronic health issues that may manifest decades after, including an increased risk of cardiovascular disease, cancer, vision impairment and neurodegenerative conditions. As the duration of exploratory missions increases and astronauts are anticipated to live for more than 20 years after returning from exploratory missions, we now propose that the development of cardiac disease and disruption of homeostasis in multiple systems caused by space radiation and weightlessness develops after extended follow-up periods.

The stress of microgravity during spaceflight or HLU dysregulates the immune system and may lead to acute and chronic health issues. The present study shows that the deleterious effects of 30 days of HLU persist 240 days after reloading, manifesting as an increase in multiple immune cells in the circulation: total leukocytes, T lymphocytes, helper T lymphocytes, cytotoxic T lymphocytes and B lymphocytes, and one immune system cell type (NK cells) that decreased in the spleen. Exposure to simGCRsim with no HLU did not change the levels of lymphoid and myeloid cells present in the circulation. Sham irradiation with HLU increased lymphocytes but not myeloid cells in blood compared with sham-irradiation without HLU that was inhibited by simGCRsim with HLU, indicating an interaction between spaceflight stressors. The spleen has important roles in hematopoiesis, red blood cell clearance, and immune function. Exposure to simGCRsim with no HLU depleted natural killer cells resident in the spleen by 67% 240 days after reloading with no further decrease with simGCRsim with HLU. NK cell function in astronauts flying 9–10 days aboard the Space Shuttle decreased upon return to 1g [39]. Exposure of mice to 0.4 Gy ^16^O ions over three fractions decreased NK cell function in the spleen by 25% after 365–548 days [40]. In contrast, combined exposure to 0.5 Gy gamma radiation and hind limb unloading for 30–31 days without reloading in mice did not alter immune cells in the spleen [41]. A selective suppression of NK cell function is a known effect of spaceflight and constitutes another of the important considerations for astronaut health during deep space missions [42,43]. Our findings show that the extent of immune cell dysregulation in the spleen caused by the spaceflight stressors simGCRsim and HLU followed by unloading is apparent later than reported in previous studies, indicating its importance to astronaut health long after the completion of exploratory missions. Together, simGCRsim and HLU did not exert a late effect on the levels of immune cells present in heart tissue, indicating the response to these spaceflight stressors is tissue specific.

The present study showed that extended reloading periods cause significant inhibition of cytokine production following hindlimb unloading. Our results confirm and extend the observation of decreased cytokine levels in the flight subject as part of the NASA Twins study [44]. HLU for 30 days persistently reduced levels of 20 of 27 (74%) cytokines in the circulation over the 270 days of reloading. In the NASA Twins Study, living on the International Space Station for 340 days decreased levels of 17 of 50 (34%) cytokines in the flight subject 190 days after return to 1g [44]. Six of the decreased cytokines, EGF, GRO/KC, Il-10, IL-18, IP10, and leptin (S4 Table) were common to rats and humans, supporting the utility of the rat as a model for weightlessness in humans. These findings suggest that simulated weightlessness using HLU in rats and actual weightlessness in humans in space each cause prolonged inhibition of cells that produce cytokines at later times after reloading. Humans live far longer than rats [45], 270 rat days = 9,396 human days = 26 years, so prolonged decreases in cytokine production observed in rats may result in chronic health issues in astronauts many years after returning to 1g. Addressing this issue is critical, given that astronauts are anticipated to live for more than two decades following the completion of exploratory missions [2] and current knowledge about chronic health outcomes is limited by the relatively small numbers of career astronauts.

We identified 7 gene variants induced by simGCRsim: *Smc3, Suz12, Brcc3, Ep300, Cblb, Idh2* and *Usaf2 [*46*–*49*]*, each of which may indicate clonal hematopoiesis. While CHIP is not an established disease, it can inform long-term risk for cardiovascular disease and cancer. The presence of indicators of clonal hematopoiesis in rats exposed to simGCRsim suggests the potential for the development of these two diseases following prolonged spaceflight, as noted in the NASA Twin Study [44]. An increase in the VAF for the *TET2* gene was present after an astronaut, 52 years of age, living aboard the International Space Station, returned to Earth [50]. With only one subject, it is not possible to determine if this VAF increase was due to time in space or changes in age. Some other increases developed over time in VAF in two separate gene variants for *DNMT3A* in the twin brother living on Earth [50]. The twin brother had also been an astronaut and may have developed VAF’s due to time in space, or each could be age-related. These findings indicate increases in VAF are not specific to spaceflight and may be induced by age alone.

In 14 astronauts with a median age of 42, who flew 12-day Space Shuttle missions, gene variants were present in *DNMT3A* and *TP53* [51]. Aging is CHIP's strongest known risk factor [14,29,52]. Our findings indicate gene variants indicative of clonal hematopoiesis arising after exposure to simGCRsim in rats are distinct from those in astronauts living in low earth orbit partially protected by the Earth’s magnetosphere. Ontological analysis reveals the effects of simGCRsim map to regions of 4 chromosomes mainly containing immune/MHC/RT1 genes, with additional smaller blocks in genes related to cell regulation, extracellular matrix signaling, DNA repair and cytoskeleton that can lead to persistent adverse health effects. We recognize that these data are for a single time point in three subjects and that further study will be needed to ascertain the significance of this observation. Our observations of variant alleles associated with CHIP in this rat study are intriguing, however, a more detailed time-course study with pre-and post-irradiation samples and longitudinal follow up throughout the observation period would provide more rigorous comparisons. CHIP has been linked to an approximately 1.9 times higher risk of coronary heart disease and a fourfold greater risk of early-onset myocardial infarction [14]. Aging is the strongest known risk factor for CHIP [14,29,52]. CHIP carriers have ~ 2x the relative risk of cardiovascular disease as non-CHIP carriers [14,29]. CHIP carriers > 50 years old have an almost 4-fold increase in cardiovascular risk. Thus, middle-aged astronauts exposed to GCR during exploratory missions are at increased risk of developing CHIP.

In the present study, exposure to the spaceflight stressors simGCRsim and HLU alone and in combination triggered systemic alterations in serum calcium levels 240 days of reloading after 30 days of HLU. However, feedback systems tightly managed these shifts, keeping calcium homeostasis stable and within the normal physiological range. In an astronaut study, exposure to microgravity has been linked to accelerated bone breakdown and increased urinary calcium loss, leading to a negative calcium balance that progressively decreases bone mineral density [53]. Even with physical and nutritional countermeasures present in astronauts during microgravity, sustained decreases in blood calcium occurred 360 days after returning to Earth [4]. In an astronaut study, parathyroid hormone levels decreased 360 days after astronauts returned to Earth. In our rat study, calcium levels decreased after exposure to 1.5 Gy simGCRsim with HLU and 1.5 Gy simGCRsim with no HLU compared with controls intermittently over the 270-day study period. The present study shows serum 25-hydroxyvitamin D_3_ decreased after exposure to simGCRsim and HLU despite inclusion of vitamin D_3_ in the standard rat chow (2.4 IU/g). Serum levels of 25-hydroxyvitamin D_3_ are a primary indicator of vitamin intake, absorption, and stores, reflecting vitamin D_3_ produced endogenously and that obtained from foods and supplements [54]. In serum, 25-hydroxyvitamin D_3_ has a half-life of 15 days [28], so the persistent decreases observed in the present study may reflect a chronic defect in vitamin D intake/stores and/or hepatic conversion of vitamin D_3_ to 25-hydroxyvitamin D_3_.

Vitamin D_3_ plays a critical role in calcium metabolism. The risk of vitamin D_3_ insufficiency or deficiency may become high for astronauts during exploratory missions because they could lack ultraviolet light exposure during transit to and from Mars and in the surface habitat, and may have an insufficient dietary supply and/or absorption of vitamin D. Four- to 6-months of space flight aboard the International Space Station decreased 25-hydroxyvitamin D_3_ by 25% from the pre-flight level of 63 ± 16 nmol/L to 48 ± 20 nmol/L, despite the provision of supplemental oral vitamin D_3_ (400 IU/day, [55]. Low serum 25-hydroxyvitamin D_3_ has been suggested as a risk factor for cardiovascular disease [56,57]. Reduced serum 25-hydroxyvitamin D_3_ has been identified during HLU in rats [58], and as with human spaceflight, calcium metabolism is known to be affected by reduced weight bearing in rodents. Further studies of calcium and vitamin D are needed to understand the underlying mechanism and to develop countermeasures to maintain calcium homeostasis during and after deep space exploratory missions.

Our study provides insight into whether synergy or antagonism exists between the two spaceflight stressors, simGCRsim and HLU, and how this impacts outcomes. Increased levels of lymphoid cells in the blood in sham-irradiated rats with HLU are abolished by simGCRsim and HLU. simGCRsim no HLU has no effect on levels of the immune cells measured. These findings suggest antagonism between the two spaceflight stressors. Exposure to 1.5 Gy of simGCRsim with or without HLU increased cardiac perivascular collagen content compared to sham-irradiated no HLU rats after 270 days. The extent of cardiac fibrosis present in the two irradiated groups could not be distinguished, indicating no interaction between the two space flight stressors. Thus, the expected interaction between the two spaceflight stressors on cardiac fibrosis was not evident in the dosing conditions employed here. Sham irradiation with HLU decreased cytokines in blood compared with sham-irradiation without HLU that was inhibited by simGCRsim with HLU, indicating an interaction between spaceflight stressors. These findings suggest a limited interaction between these two spaceflight stressors on the immune system, cardiac perivascular fibrosis, and cytokines that require further study.

There are limitations to our study and its interpretation. The time course of tissue (heart and spleen) and blood (immune cells) changes after simGCRsim, and HLU was not determined. Future studies are needed to determine how these risks will change (e.g., cyclical, linear or exponential) the immune system over time. The genomic analysis is preliminary and conducted at one time point in a limited number of animals and without baseline samples on individual subjects prior to any exposures. There was no gene sequencing for HLU as this does not alter the fundamental DNA sequence in rats -its effects are epigenetic and transcriptomic [59,60]. In the NASA Twins Study, a specific group of genes—totaling 811 across multiple cell types—failed to revert to their preflight expression levels within six months after return to Earth. Many of these genes are involved in immune responses and DNA repair, suggesting that long-duration spaceflight may induce prolonged alterations in gene regulation [44]. These findings suggest astronauts conducting exploratory class missions could experience risks for immunological stress [44]. The five-ion six-beam simGCRsim incompletely represents the GCR spectrum to which astronauts will be exposed during exploratory missions. A 33-ion sequential beam galactic cosmic ray analog is now available for future studies as warranted [3]. In the present study, simGCRsim was delivered as a single acute exposure at the highest dose rates achievable. This experimental design was chosen for logistical feasibility however, it does not fully replicate the chronic, low-dose rate environment experienced by astronauts during long-duration deep space missions. The biological consequences of radiation are highly dependent on the temporal distribution of the dose. In the laboratory setting, the delivery of the total dose within minutes precludes the influence of real-time DNA repair mechanisms and cellular recovery processes that occur during protracted exposures, as well as the possibility that some DNA repair mechanisms may fail to be engaged at very low dose-rates. Acute models may fail to capture persistent signaling or chronic inflammation that would occur during an extended exploration mission. To make our findings more relevant to astronaut health, future studies on dose fractionation are needed to determine if this would affect cardiovascular outcomes.

In our prior study in WAG/RijCmcr rats, we found that whole body irradiation with 1.5 Gy of a three-ion beam grouping did not result in cardiac mechanical function using echocardiography [2]. Based on these findings, we elected not to conduct a functional cardiac assessment for the current study. Thus, cardiac perivascular fibrosis was the only direct measure of cardiac structure in the current study. Furthermore, the present study did not evaluate other cytokines, immune cells, or organs besides the heart and spleen. Last, the present study did not address the challenge of countermeasure development capable of protecting astronauts from the adverse effects of prolonged exposure to space radiation and microgravity. The findings from the present study provide the rationale for future studies to address these limitations. Of note, the development of cardiac fibrosis, a hallmark of radiation injury, after exposure to components of space radiation, has been observed in the mouse after 22 months [61], but not in the Long Evans rat after 12 months [62], mouse after 9 months [11], and rabbit after 12 months [63].

In addition to rodent models, a larger higher order animal model is needed to investigate the combined effects of HLU and simGCRsim on the risk for chronic cellular dysfunction in multiple biological systems including cardiovascular, central nervous and immune systems and vision. Minipigs are a well characterized animal model with anatomical, physiological and biochemical similarities to humans. Their size also allows for studies on dose depth relationships and volume effects to help understand radiation exposure in astronauts.

The astronaut corps includes both men and women; however, the current investigation evaluated radiation effects only in male rats. According to a policy issued by the ICRP in 2007, the use of sex-specific data is not advised for general radiological protection of occupationally exposed workers [64]. More recently, NASA requested that the National Academy of Sciences examine its radiation protection framework for deep space exploration [65]. In 2022, NASA revised its allowable spaceflight radiation exposure limits, applying the same standards across all ages and sexes [66]. Under these non-cancer risk guidelines, no modifications are made based on an astronaut’s sex or age. To better inform and validate these guidelines, future research should include studies in female rats and directly compare the outcomes with those observed in males. Follow-up studies are needed to explain the increase in immune cells in blood caused by HLU associated with no effect on immune cells in heart and the decrease in NK cells in spleen.

Space radiation-induced bystander effects occur when cells directly hit by high-linear energy transfer GCR or SPE transmit distress signals to neighboring or even distant cells and tissues that were not hit with GCR [67]. This signaling may allow healthy, non-irradiated cells to exhibit DNA damage, mutations, and genomic instability. Bystander effects can significantly increase the total volume of biological tissue affected during long-duration space flight beyond what direct particle hits alone would reflect [68]. High-LET particles like iron and silicon, present in simGCRsim, trigger significantly more severe, long lasting bystander damage than protons, and may increase health risks for astronauts conducting exploratory missions [69,70]. It is conceivable that bystander effects may transform a “local” hit from simGCRsim into a “systemic” stress response within the heart tissue or other relevant tissues, potentially increasing the overall cardiovascular risk for long-duration missions. Future studies are needed to define the extent that exposure to limiting, low fluences of simGCRsim may cause tissue level responses relevant to exploratory missions.

The present study shows the spaceflight stressors HLU and simGCRsim adversely impact multiple biological systems in the rat resulting in chronic cellular dysfunction that persists long after their discontinuation. Radiation results in cardiac perivascular disease at an exploratory mission relevant dose and alters the sequence of the genome. Simulated microgravity results in chronic activation of the immune system, and dysregulation of cytokines. Our findings are relevant to human space exploration as the effects of microgravity and galactic cosmic radiation may persist long after astronauts return to earth. Their impact on astronaut health needs to be understood prior to conducting deep space exploratory missions.

## Supporting information

S1 Fig Body weights. Body weights 19 days prior to the start of the study and 260 days after the start of the study. Data are means ± SD, *n* = 11–12 animals per group. Statistical analysis was conducted using a t-test.
(PDF)

S2 Fig Serum albumin levels. Comparison of group differences in blood albumin levels affected by HLU and simGCRsim over time. Data represents the mean ± SE, *n* = 12/group.
(PDF)

S3 Fig Biomarkers of calcium metabolism. Comparison of group differences in blood parathyroid hormone, osteocalcin, fibroblast growth factor 23 and inorganic phosphorus levels affected by HLU and simGCRsim over time. Data represent the mean ± SE, *n* = 3–6/group. Statistical analysis was conducted using a t-test.
(PDF)

S4 Fig Components of the immune system in the spleen at 270 days after simGCRsim and HLU. Rats were nine months of age at the start of the study. T cells (CD3^+^), macrophages (CD68^+^) and B cells (CD20^+^) appear brown against the blue and white background due to the staining protocols. The horizontal scale bar represents 500 microns. Images are representative of groups of 3 animals.
(PDF)

S5 Fig Risk factors for cardiac disease and kidney injury after irradiation of rats with simGCRsim no HLU. Absolute values for total cholesterol (upper left), triglycerides (upper right), blood urea nitrogen (lower left) and total protein (lower right) following sham irradiation no HLU compared with simGCRsim 1.5 Gy no HLU. Data are means ± SD, *n* = 11–12/group. * = p < 0.05 vs. age-matched control. Statistical analysis was conducted using a t-test.
(PDF)

S6 Fig Risk factors for cardiac disease and kidney injury after HLU in sham irradiated rats. Absolute values for total cholesterol (upper left), triglycerides (upper right), blood urea nitrogen (lower left) and total protein (lower right) following sham irradiation no HLU compared with sham-irradiation with HLU. Data are means ± SD, *n* = 11–12/group. * = p < 0.05 vs. age-matched, sham-irradiated control. ** = p < 0.01 vs. age-matched control. Statistical analysis was conducted using a t-test.
(PDF)

S7 Fig Risk factors for cardiac disease and kidney injury after irradiation of rats with simGCRsim with HLU. Absolute values for total cholesterol (upper left), triglycerides (upper right), blood urea nitrogen (lower left) and total protein (lower right) following irradiation with 1.5 Gy with HLU compared with sham-irradiation no HLU. Data are means ± SD, *n* = 11–12/group. * = p < 0.05 vs. age-matched control. ** = p < 0.01 vs. age-matched control. *** = p < 0.001 vs. age-matched control. Statistical analysis was conducted using a t-test.
(PDF)

S1 Table Impact of simGCRsim and HLU on cytokine release. (PDF)

S2 Table Gene variant allele frequency and additional metrics. (PDF)

S3 Table Comparison of all CHIP variants. (PDF)

S4 Table Cytokine changes common to rats and humans. (PDF)
