# Supplementary material for: Multiple simulated spaceflight stressors impact cardiac fibrosis, calcium dynamics, immune function, cytokines and gene variants in rat, Rattus norvegicus
Source: PLoS One. 2026 Sep 30;21(9):e0357485. doi: 10.1371/journal.pone.0357485 (PMC13626364; doi:10.1371/journal.pone.0357485)
Supplement: Supplementary file 1 [file pone.0357485.s001.pdf]

Figure S1

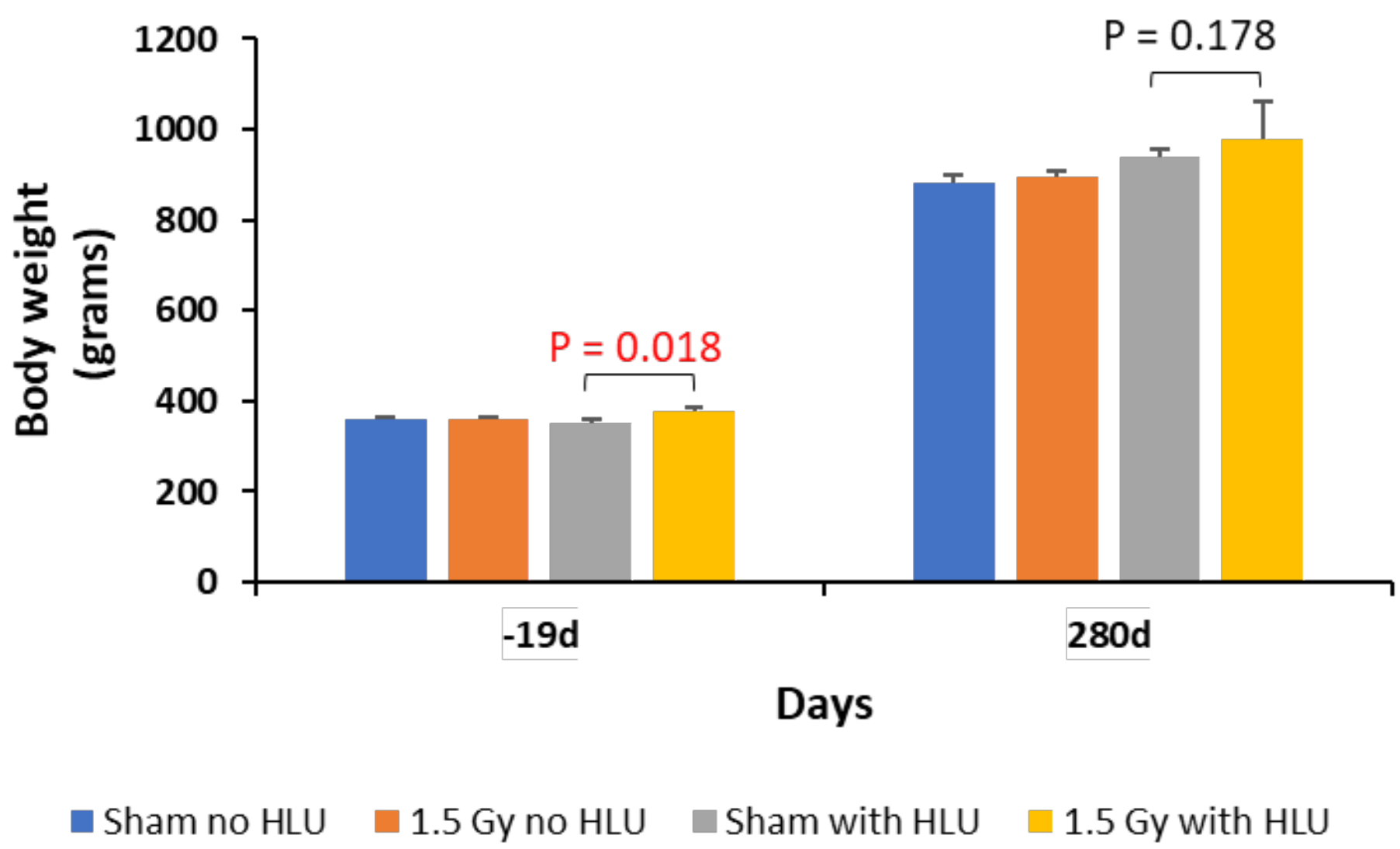

1200
1000
800
600
400
200
0
Body weight (grams)
P = 0.018
P = 0.178
-19d
280d
Days
Sham no HLU
1.5 Gy no HLU
Sham with HLU
1.5 Gy with HLU
