# Supplementary material for: Multiple simulated spaceflight stressors impact cardiac fibrosis, calcium dynamics, immune function, cytokines and gene variants in rat, Rattus norvegicus
Source: PLoS One. 2026 Sep 30;21(9):e0357485. doi: 10.1371/journal.pone.0357485 (PMC13626364; doi:10.1371/journal.pone.0357485)
Supplement: Supplementary file 2 [file pone.0357485.s002.pdf]

Figure S2

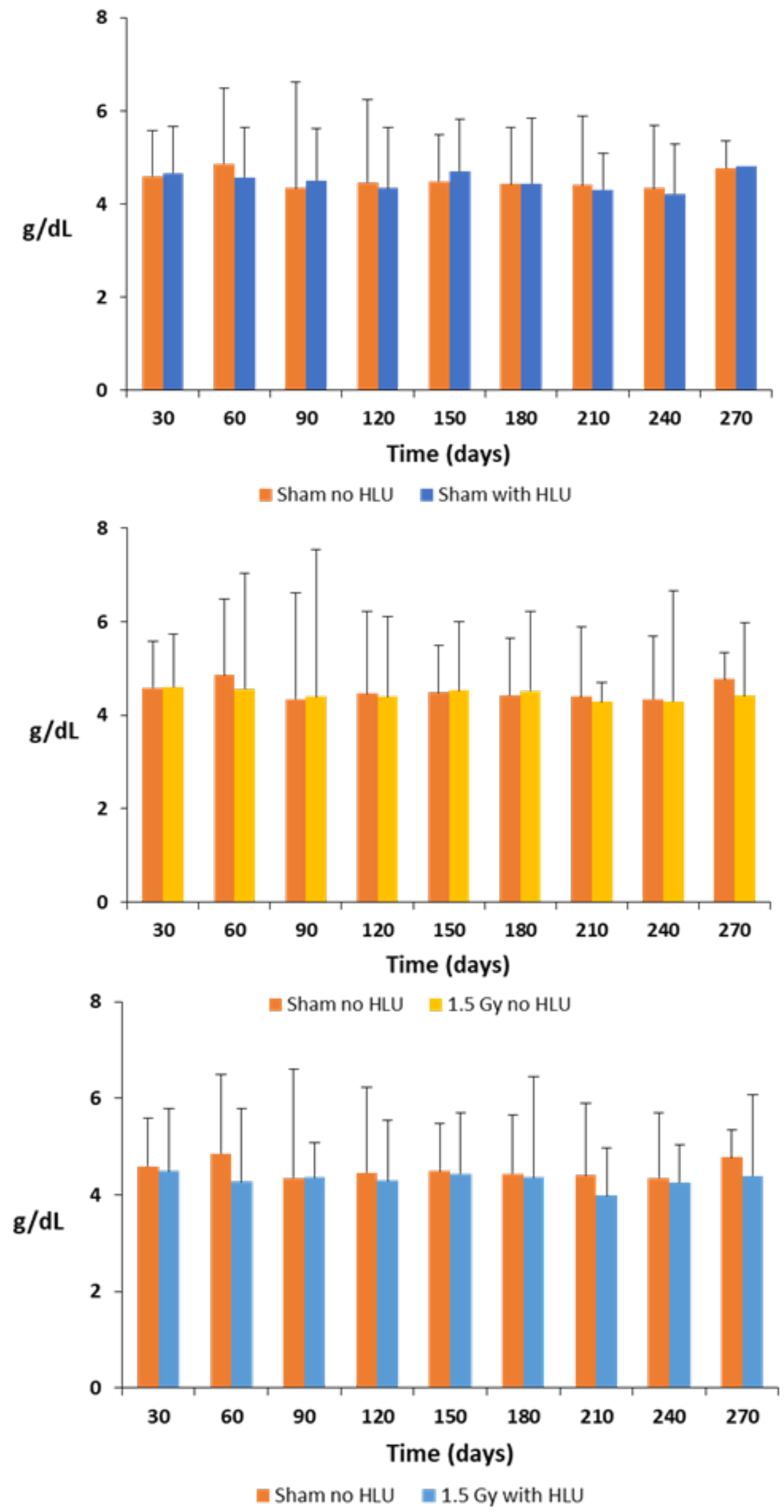

g/dL
0
2
4
6
8
30
60
90
120
150
180
210
240
270
Time (days)
Sham no HLU
Sham with HLU
g/dL
0
2
4
6
8
30
60
90
120
150
180
210
240
270
Time (days)
Sham no HLU
1.5 Gy no HLU
g/dL
0
2
4
6
8
30
60
90
120
150
180
210
240
270
Time (days)
Sham no HLU
1.5 Gy with HLU
