# Supplementary material for: Multiple simulated spaceflight stressors impact cardiac fibrosis, calcium dynamics, immune function, cytokines and gene variants in rat, Rattus norvegicus
Source: PLoS One. 2026 Sep 30;21(9):e0357485. doi: 10.1371/journal.pone.0357485 (PMC13626364; doi:10.1371/journal.pone.0357485)
Supplement: Supplementary file 3 [file pone.0357485.s003.pdf]

Figure S3

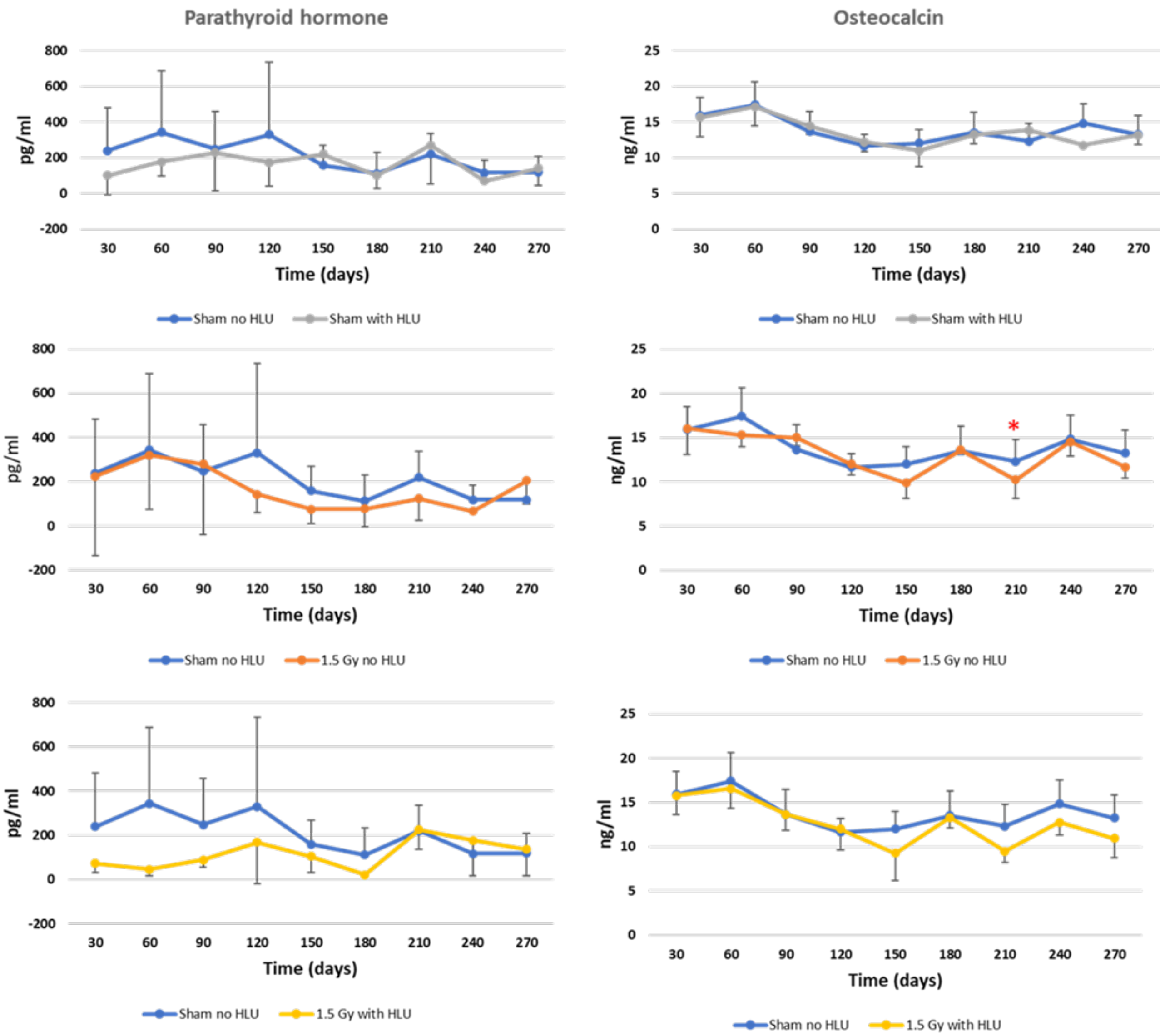

Parathyroid hormone
Osteocalcin
pg/ml
ng/ml
Time (days)
30
60
90
120
150
180
210
240
270
Sham no HLU
Sham with HLU
1.5 Gy no HLU
1.5 Gy with HLU
*

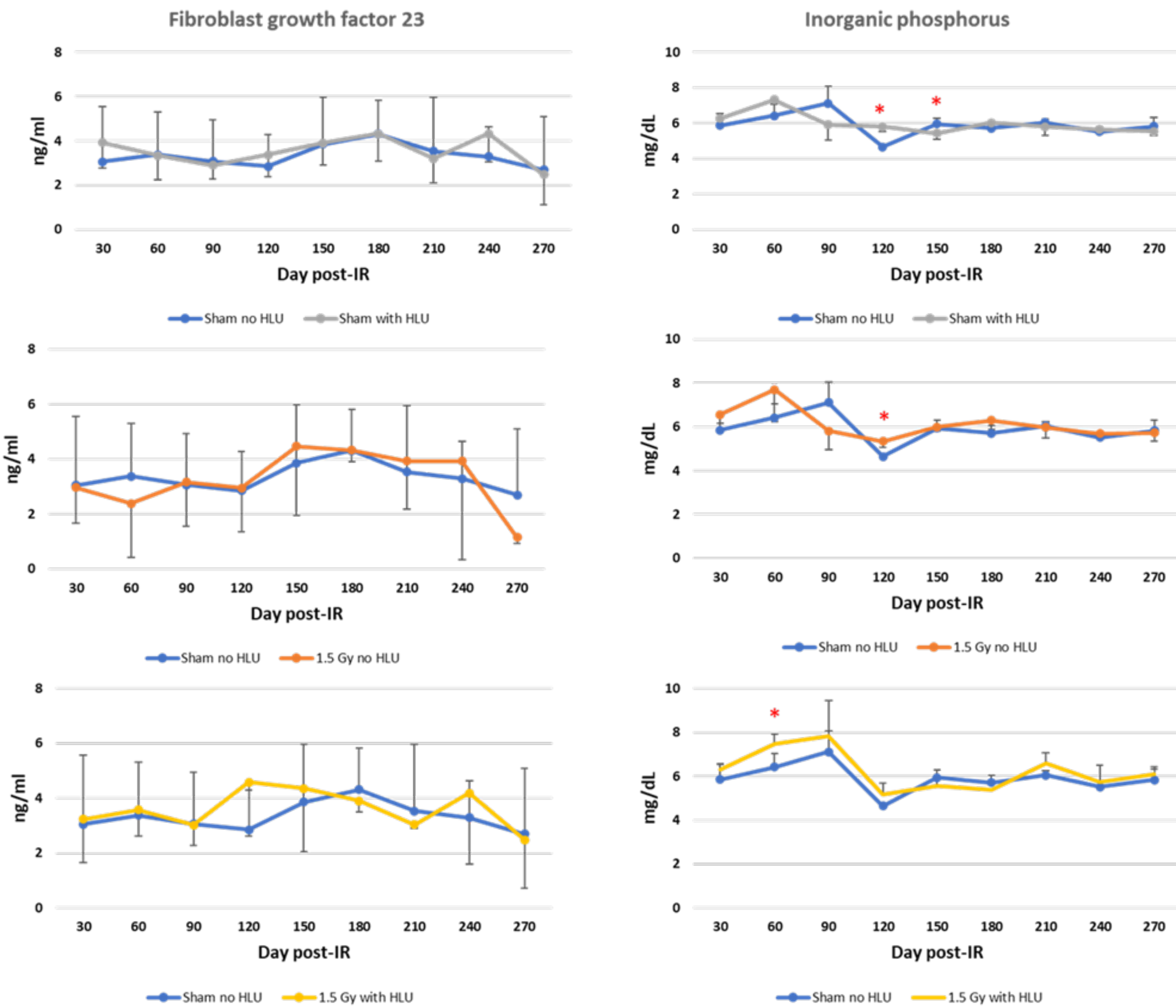
Fibroblast growth factor 23
Inorganic phosphorus
ng/ml
mg/dL
30 60 90 120 150 180 210 240 270
Day post-IR
Sham no HLU
Sham with HLU
1.5 Gy no HLU
1.5 Gy with HLU
*
