# Supplementary material for: Multiple simulated spaceflight stressors impact cardiac fibrosis, calcium dynamics, immune function, cytokines and gene variants in rat, Rattus norvegicus
Source: PLoS One. 2026 Sep 30;21(9):e0357485. doi: 10.1371/journal.pone.0357485 (PMC13626364; doi:10.1371/journal.pone.0357485)
Supplement: Supplementary file 4 [file pone.0357485.s004.pdf]

Figure S4

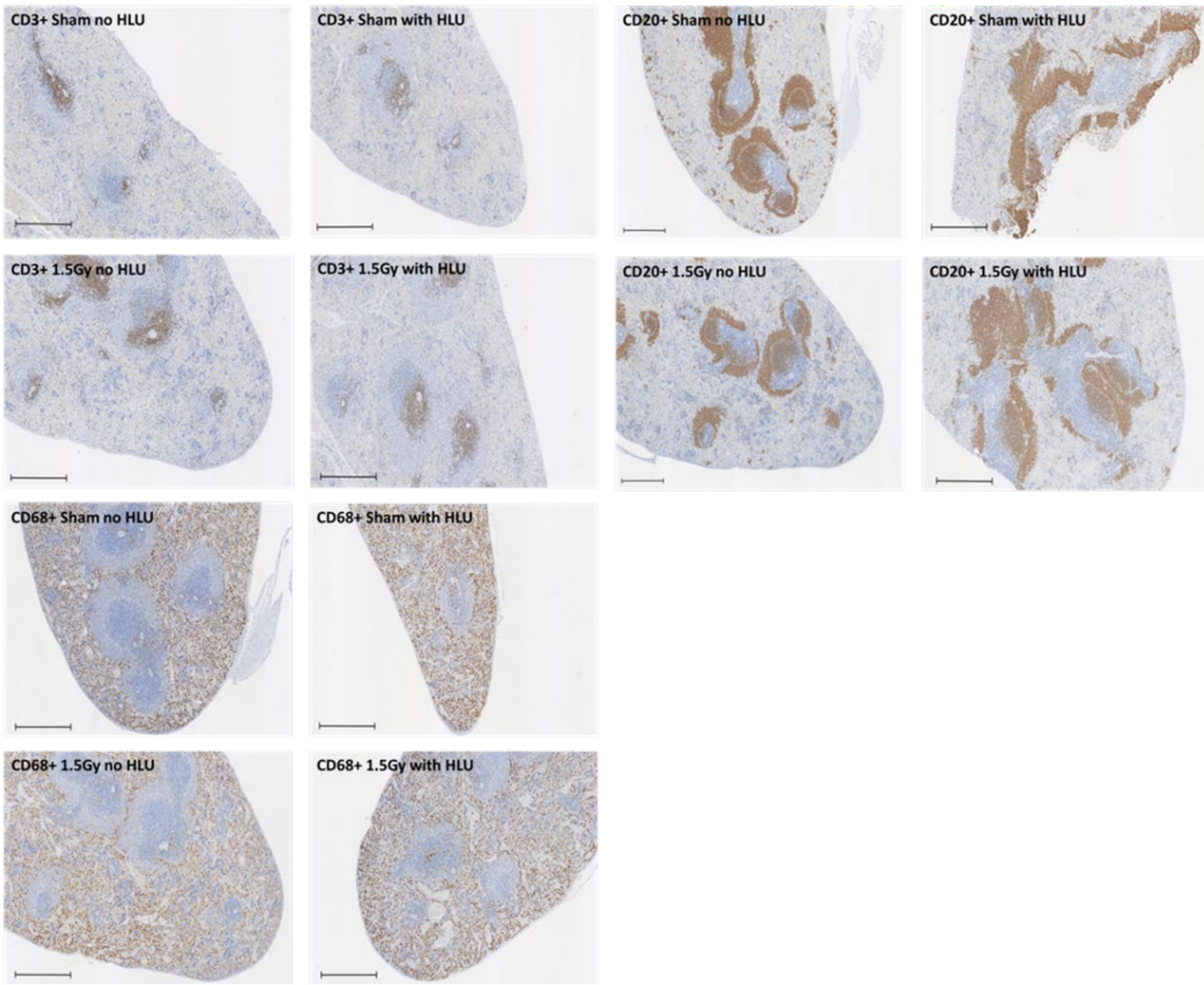
CD3+ Sham no HLU
CD3+ Sham with HLU
CD20+ Sham no HLU
CD20+ Sham with HLU
CD3+ 1.5Gy no HLU
CD3+ 1.5Gy with HLU
CD20+ 1.5Gy no HLU
CD20+ 1.5Gy with HLU
CD68+ Sham no HLU
CD68+ Sham with HLU
CD68+ 1.5Gy no HLU
CD68+ 1.5Gy with HLU
