# Supplementary material for: Multiple simulated spaceflight stressors impact cardiac fibrosis, calcium dynamics, immune function, cytokines and gene variants in rat, Rattus norvegicus
Source: PLoS One. 2026 Sep 30;21(9):e0357485. doi: 10.1371/journal.pone.0357485 (PMC13626364; doi:10.1371/journal.pone.0357485)
Supplement: Supplementary file 6 [file pone.0357485.s006.pdf]

Figure S6

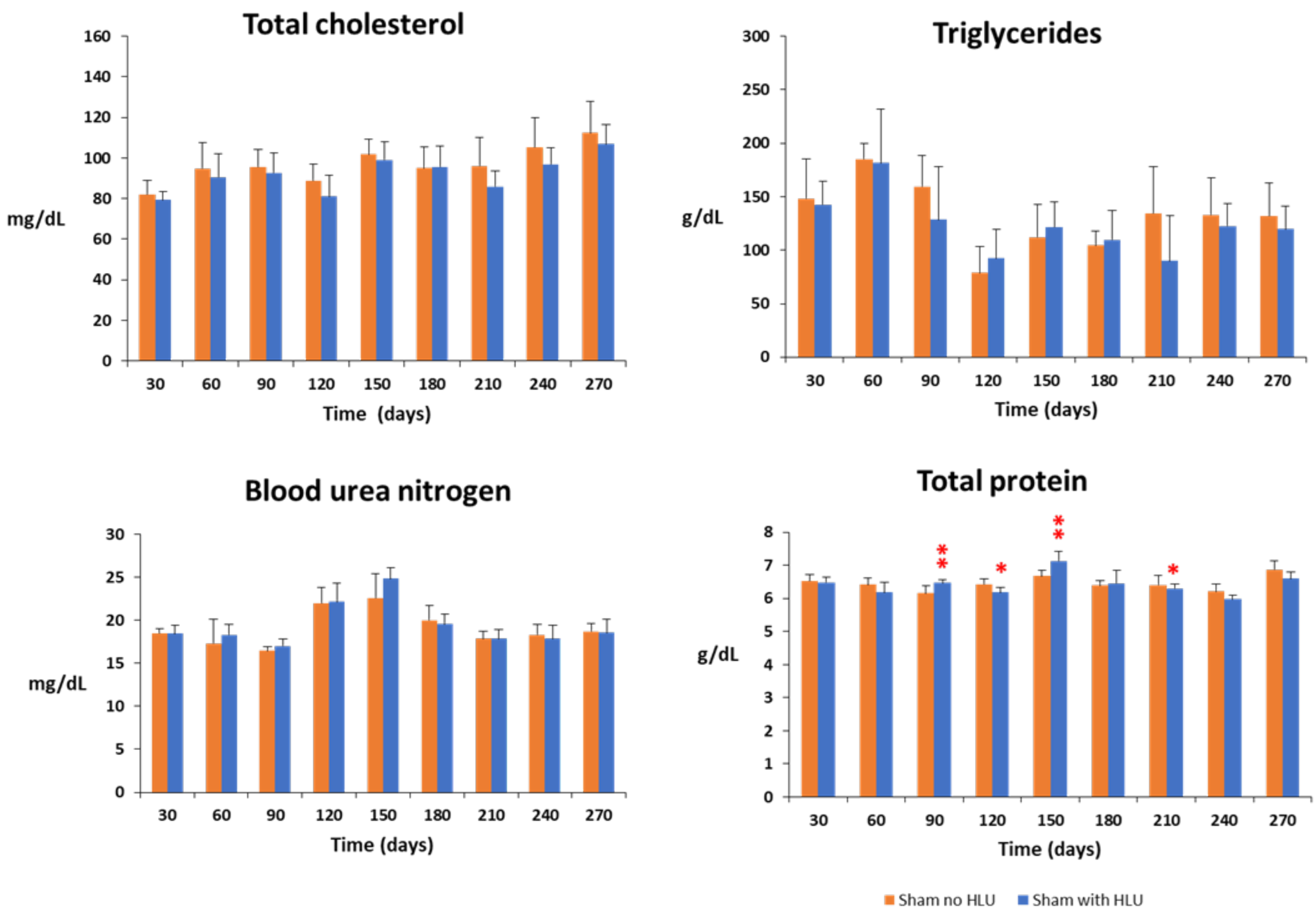
Total cholesterol
mg/dL
0 20 40 60 80 100 120 140 160
30 60 90 120 150 180 210 240 270
Time (days)
Triglycerides
g/dL
0 50 100 150 200 250 300
30 60 90 120 150 180 210 240 270
Time (days)
Blood urea nitrogen
mg/dL
0 5 10 15 20 25 30
30 60 90 120 150 180 210 240 270
Time (days)
Total protein
g/dL
0 1 2 3 4 5 6 7 8
**
*
**
*
30 60 90 120 150 180 210 240 270
Time (days)
Sham no HLU
Sham with HLU
