# Supplementary material for: Multiple simulated spaceflight stressors impact cardiac fibrosis, calcium dynamics, immune function, cytokines and gene variants in rat, Rattus norvegicus
Source: PLoS One. 2026 Sep 30;21(9):e0357485. doi: 10.1371/journal.pone.0357485 (PMC13626364; doi:10.1371/journal.pone.0357485)
Supplement: Supplementary file 7 [file pone.0357485.s007.pdf]

Figure S7

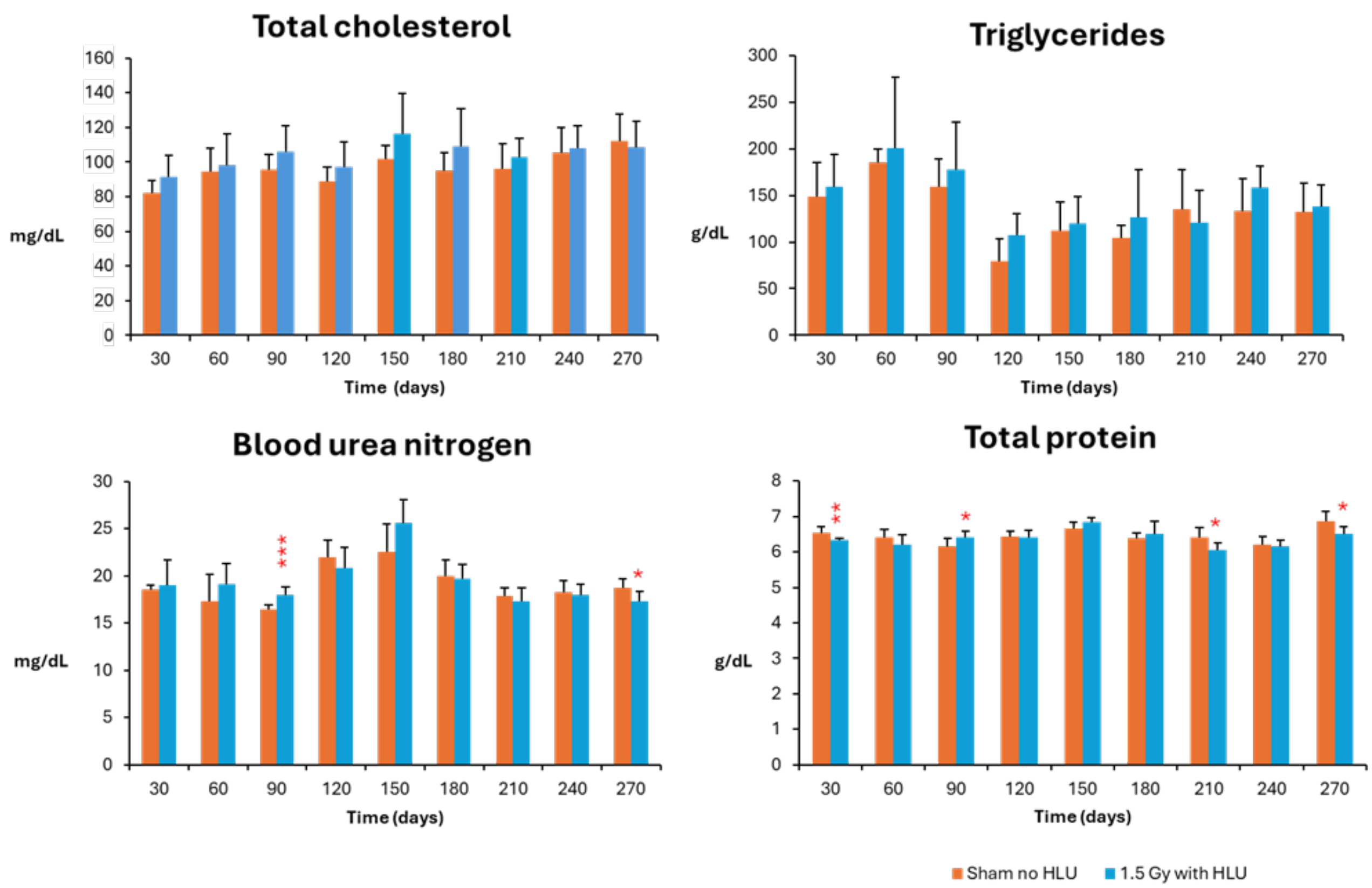

Total cholesterol
mg/dL
160
140
120
100
80
60
40
20
0
30 60 90 120 150 180 210 240 270
Time (days)
Triglycerides
g/dL
300
250
200
150
100
50
0
30 60 90 120 150 180 210 240 270
Time (days)
Blood urea nitrogen
mg/dL
30
25
20
15
10
5
0
30 60 90 120 150 180 210 240 270
Time (days)
Total protein
g/dL
8
7
6
5
4
3
2
1
0
30 60 90 120 150 180 210 240 270
Time (days)
Sham no HLU
1.5 Gy with HLU
