# Supplementary material for: Multiple simulated spaceflight stressors impact cardiac fibrosis, calcium dynamics, immune function, cytokines and gene variants in rat, Rattus norvegicus
Source: PLoS One. 2026 Sep 30;21(9):e0357485. doi: 10.1371/journal.pone.0357485 (PMC13626364; doi:10.1371/journal.pone.0357485)
Supplement: Supplementary file 8 [file pone.0357485.s008.pdf]

Supplement Table 1.

Sham with HLU vs. Sham no HLU

outcome	contrast	ratio	lower.CL	upper.CL	p.value	q
**EGF**	**Sham with HLU / Sham no HLU**	**0.5641**	**0.3504**	**0.9082**	**0.0207**	**0.0620**
**Eotaxin**	**Sham with HLU / Sham no HLU**	**0.8862**	**0.7782**	**1.0091**	**0.0667**	**0.0934**
**Fractalkine**	**Sham with HLU / Sham no HLU**	**0.7290**	**0.5173**	**1.0273**	**0.0692**	**0.0934**
G CSF	Sham with HLU / Sham no HLU	0.8635	0.6691	1.1144	0.2455	0.2651
**GM_CSF**	**Sham with HLU / Sham no HLU**	**0.7662**	**0.6158**	**0.9533**	**0.0192**	**0.0620**
GRO_KC	Sham with HLU / Sham no HLU	0.8582	0.7448	0.9888	0.0356	0.0642
IFN gamma	Sham with HLU / Sham no HLU	0.8095	0.6519	1.0052	0.0552	0.0829
IL_1alpha	Sham with HLU / Sham no HLU	0.7983	0.6518	0.9778	0.0312	0.0642
IL_1beta	Sham with HLU / Sham no HLU	0.8214	0.6826	0.9886	0.0384	0.0649
IL_2	Sham with HLU / Sham no HLU	0.6889	0.5096	0.9315	0.0177	0.0620
IL_4	Sham with HLU / Sham no HLU	0.8542	0.7421	0.9833	0.0299	0.0642
IL_5	Sham with HLU / Sham no HLU	0.9316	0.8507	1.0202	0.1201	0.1474
IL_6	Sham with HLU / Sham no HLU	0.8016	0.6577	0.9769	0.0301	0.0642
IL_10	Sham with HLU / Sham no HLU	0.8027	0.6468	0.9961	0.0463	0.0735
IL_12p70	Sham with HLU / Sham no HLU	0.7746	0.6654	0.9018	0.0021	0.0575
IL_13	Sham with HLU / Sham no HLU	0.8680	0.7208	1.0453	0.1284	0.1507
IL 17A	Sham with HLU / Sham no HLU	0.8574	0.7161	1.0265	0.0902	0.1159
IL_18	Sham with HLU / Sham no HLU	0.7087	0.5190	0.9678	0.0318	0.0642
IP_10	Sham with HLU / Sham no HLU	0.8983	0.8368	0.9643	0.0048	0.0620
Leptin	Sham with HLU / Sham no HLU	0.7290	0.5799	0.9164	0.0089	0.0620
LIX	Sham with HLU / Sham no HLU	0.8510	0.6183	1.1713	0.3072	0.3190

MCP_ 1	Sham with HLU / Sham no HLU	0.9498	0.8006	1.1269	0.5386	0.5386
MIP_1alpha	Sham with HLU / Sham no HLU	0.7372	0.5828	0.9325	0.0133	0.0620
**MIP_2**	Sham with HLU / Sham no HLU	0.8471	0.7453	0.9629	0.0136	0.0620
RANTES	Sham with HLU / Sham no HLU	0.8956	0.8185	0.9799	0.0187	0.0620
TNFalpha	Sham with HLU / Sham no HLU	0.8843	0.7234	1.0809	0.2171	0.2442
VEGF	Sham with HLU / Sham no HLU	0.5472	0.3151	0.9501	0.0336	0.0642

0.75 Gy no HLU vs. Sham no HLU and 1.5Gy no HLU vs. Sham no HLU

outcome	contrast	ratio	lower.CL	upper.CL	p.value	q
EGF	0.75Gy no HLU / Sham no HLU	0.9263	0.6030	1.4229	0.7149	0.8042
Eotaxin	0.75Gy no HLU / Sham no HLU	0.8861	0.7713	1.0179	0.0842	0.6080
Fractalkine	0.75Gy no HLU / Sham no HLU	0.8956	0.6355	1.2623	0.5129	0.7318
G CSF	0.75Gy no HLU / Sham no HLU	0.9069	0.7139	1.1521	0.4058	0.7318
GM CSF	0.75Gy no HLU / Sham no HLU	0.8248	0.6583	1.0334	0.0901	0.6080
GRO KC	0.75Gy no HLU / Sham no HLU	0.8917	0.7806	1.0187	0.0877	0.6080
IFN gamma	0.75Gy no HLU / Sham no HLU	0.8778	0.7375	1.0448	0.1345	0.6231
IL_1alpha	0.75Gy no HLU / Sham no HLU	0.8577	0.6991	1.0523	0.1335	0.6231
IL 1beta	0.75Gy no HLU / Sham no HLU	0.9296	0.7571	1.1413	0.4678	0.7318
IL 2	0.75Gy no HLU / Sham no HLU	0.9390	0.7147	1.2337	0.6374	0.7936
IL 4	0.75Gy no HLU / Sham no HLU	0.9599	0.8369	1.1011	0.5421	0.7318
IL 5	0.75Gy no HLU / Sham no HLU	0.9553	0.8718	1.0468	0.3108	0.7318
IL 6	0.75Gy no HLU / Sham no HLU	0.8279	0.6721	1.0197	0.0733	0.6080
IL 10	0.75Gy no HLU / Sham no HLU	0.9010	0.7095	1.1443	0.3751	0.7318
IL_12p70	0.75Gy no HLU / Sham no HLU	0.9214	0.7848	1.0819	0.3015	0.7318
IL 13	0.75Gy no HLU / Sham no HLU	0.8694	0.7179	1.0527	0.1432	0.6231
IL 17A	0.75Gy no HLU / Sham no HLU	0.8981	0.7698	1.0477	0.1616	0.6231
IL 18	0.75Gy no HLU / Sham no HLU	0.9092	0.6728	1.2287	0.5191	0.7318
IP_10	0.75Gy no HLU / Sham no HLU	0.9402	0.8619	1.0255	0.1551	0.6231
Leptin	0.75Gy no HLU / Sham no HLU	0.9275	0.7531	1.1424	0.4623	0.7318
LIX	0.75Gy no HLU / Sham no HLU	1.0001	0.7928	1.2616	0.9995	0.9995
MCP 1	0.75Gy no HLU / Sham no HLU	0.9363	0.7770	1.1282	0.4715	0.7318
MIP_1alpha	0.75Gy no HLU / Sham no HLU	0.9610	0.7755	1.1909	0.7042	0.8042

outcome	contrast	ratio	lower.CL	upper.CL	p.value	q
**MIP 2**	0.75Gy no HLU / Sham no HLU	0.9160	0.8038	1.0438	0.1769	0.6367
RANTES	0.75Gy no HLU / Sham no HLU	0.9101	0.8244	1.0047	0.0608	0.6080
TNFalpha	0.75Gy no HLU / Sham no HLU	0.9213	0.7097	1.1959	0.5213	0.7318
VEGF	0.75Gy no HLU / Sham no HLU	1.0388	0.6808	1.5849	0.8537	0.9040
EGF	1.5Gy no HLU / Sham no HLU	0.9505	0.6204	1.4563	0.8075	0.8721
Eotaxin	1.5Gy no HLU / Sham no HLU	0.9669	0.8697	1.0749	0.5158	0.7318
Fractalkine	1.5Gy no HLU / Sham no HLU	0.8564	0.6016	1.2192	0.3730	0.7318
G CSF	1.5Gy no HLU / Sham no HLU	0.9956	0.7666	1.2930	0.9724	0.9908
GM CSF	1.5Gy no HLU / Sham no HLU	0.9133	0.7454	1.1192	0.3646	0.7318
GRO KC	1.5Gy no HLU / Sham no HLU	0.9438	0.8304	1.0727	0.3588	0.7318
IFN gamma	1.5Gy no HLU / Sham no HLU	0.9174	0.7489	1.1239	0.3877	0.7318
IL_1alpha	1.5Gy no HLU / Sham no HLU	0.9399	0.7710	1.1458	0.5229	0.7318
IL 1beta	1.5Gy no HLU / Sham no HLU	0.9209	0.7544	1.1241	0.4003	0.7318
IL 2	1.5Gy no HLU / Sham no HLU	0.9128	0.6783	1.2283	0.5305	0.7318
IL 4	1.5Gy no HLU / Sham no HLU	0.9485	0.8136	1.1057	0.4815	0.7318
IL 5	1.5Gy no HLU / Sham no HLU	0.9319	0.8028	1.0818	0.3375	0.7318
IL 6	1.5Gy no HLU / Sham no HLU	0.9610	0.7979	1.1574	0.6615	0.7939
IL_10	1.5Gy no HLU / Sham no HLU	0.9462	0.7524	1.1898	0.6211	0.7936
IL_12p70	1.5Gy no HLU / Sham no HLU	0.8892	0.7631	1.0360	0.1251	0.6231
IL 13	1.5Gy no HLU / Sham no HLU	0.8514	0.6324	1.1461	0.2738	0.7318
IL 17A	1.5Gy no HLU / Sham no HLU	0.9302	0.7867	1.0999	0.3803	0.7318
IL_18	1.5Gy no HLU / Sham no HLU	0.8894	0.6529	1.2114	0.4400	0.7318
IP 10	1.5Gy no HLU / Sham no HLU	0.8855	0.8194	0.9570	0.0037	0.1002
Leptin	1.5Gy no HLU / Sham no HLU	0.7187	0.5878	0.8788	0.0025	0.1002
**LIX**	1.5Gy no HLU / Sham no HLU	0.9612	0.7575	1.2198	0.7345	0.8094

outcome	contrast	ratio	lower.CL	upper.CL	p.value	q
MCP 1	1.5Gy no HLU / Sham no HLU	1.0427	0.8873	1.2254	0.5960	0.7850
MIP_1alpha	1.5Gy no HLU / Sham no HLU	0.9490	0.7514	1.1986	0.6467	0.7936
MIP 2	1.5Gy no HLU / Sham no HLU	0.9753	0.8591	1.1073	0.6865	0.8042
RANTES	1.5Gy no HLU / Sham no HLU	0.8594	0.7710	0.9579	0.0084	0.1517
TNFalpha	1.5Gy no HLU / Sham no HLU	1.0674	0.8599	1.3251	0.5375	0.7318
VEGF	1.5Gy no HLU / Sham no HLU	1.0129	0.6476	1.5844	0.9532	0.9899

1.5 Gy with HLU vs. Sham no HLU

outcome	contrast	ratio	lower.CL	upper.CL	p.value	q
EGF	1.5Gy with HLU / Sham no HLU	1.0925	0.7619	1.5665	0.6158	0.9204
Eotaxin	1.5Gy with HLU / Sham no HLU	0.9738	0.8558	1.1079	0.6732	0.9204
Fractalkine	1.5Gy with HLU / Sham no HLU	0.9285	0.6634	1.2995	0.6524	0.9204
G_CSF	1.5Gy with HLU / Sham no HLU	0.9326	0.7036	1.2362	0.6131	0.9204
GM CSF	1.5Gy with HLU / Sham no HLU	0.8981	0.7210	1.1188	0.3210	0.9204
GRO KC	1.5Gy with HLU / Sham no HLU	0.9526	0.8163	1.1117	0.5211	0.9204
IFN gamma	1.5Gy with HLU / Sham no HLU	0.9167	0.7637	1.1005	0.3345	0.9204
IL_1alpha	1.5Gy with HLU / Sham no HLU	0.9655	0.7845	1.1884	0.7293	0.9204
IL 1beta	1.5Gy with HLU / Sham no HLU	1.0345	0.8865	1.2072	0.6533	0.9204
IL 2	1.5Gy with HLU / Sham no HLU	0.9972	0.7662	1.2980	0.9828	0.9828
IL 4	1.5Gy with HLU / Sham no HLU	1.0158	0.8836	1.1676	0.8182	0.9204
IL_5	1.5Gy with HLU / Sham no HLU	0.9743	0.8744	1.0855	0.6224	0.9204
IL 6	1.5Gy with HLU / Sham no HLU	0.9318	0.7395	1.1741	0.5328	0.9204
IL 10	1.5Gy with HLU / Sham no HLU	1.0361	0.8413	1.2760	0.7272	0.9204
IL_12p70	1.5Gy with HLU / Sham no HLU	0.7642	0.6227	0.9378	0.0123	0.3331
IL 13	1.5Gy with HLU / Sham no HLU	0.9291	0.7483	1.1536	0.4885	0.9204

outcome	contrast	ratio	lower.CL	upper.CL	p.value	q
IL 17A	1.5Gy with HLU / Sham no HLU	0.9401	0.7671	1.1521	0.5355	0.9204
IL 18	1.5Gy with HLU / Sham no HLU	0.9617	0.7165	1.2907	0.7858	0.9204
IP 10	1.5Gy with HLU / Sham no HLU	0.9919	0.9265	1.0620	0.8076	0.9204
Leptin	1.5Gy with HLU / Sham no HLU	0.8571	0.7067	1.0394	0.1116	0.9204
**LIX**	1.5Gy with HLU / Sham no HLU	0.8802	0.6557	1.1816	0.3797	0.9204
**MCP_** 1	1.5Gy with HLU / Sham no HLU	0.9729	0.8263	1.1455	0.7303	0.9204
MIP_1alpha	1.5Gy with HLU / Sham no HLU	0.9927	0.8066	1.2218	0.9425	0.9788
MIP 2	1.5Gy with HLU / Sham no HLU	0.9640	0.8469	1.0973	0.5631	0.9204
RANTES	1.5Gy with HLU / Sham no HLU	0.9556	0.8477	1.0772	0.4397	0.9204
TNFalpha	1.5Gy with HLU / Sham no HLU	1.0111	0.8106	1.2614	0.9181	0.9788
VEGF	1.5Gy with HLU / Sham no HLU	1.0863	0.7257	1.6260	0.6752	0.9204

1.5 Gy with HLU vs. Sham with HLU

outcome	contrast	ratio	lower.CL	upper.CL	p.value	q
**EGF**	**1.5Gy with HLU / Sham with HLU**	**1.9417**	**1.3549**	**2.7827**	**0.0009**	**0.0169**
Eotaxin	1.5Gy with HLU / Sham with HLU	1.1010	0.9588	1.2644	0.1632	0.2491
**Fractalkine**	**1.5Gy with HLU / Sham with HLU**	**1.2815**	**1.0791**	**1.5220**	**0.0067**	**0.0303**
G CSF	1.5Gy with HLU / Sham with HLU	1.0802	0.8123	1.4364	0.5802	0.6527
GM CSF	1.5Gy with HLU / Sham with HLU	1.1698	0.9443	1.4490	0.1429	0.2491
GRO KC	1.5Gy with HLU / Sham with HLU	1.1150	0.9758	1.2741	0.1045	0.2015
IFN gamma	1.5Gy with HLU / Sham with HLU	1.1350	0.8891	1.4488	0.2937	0.4173
IL_1alpha	1.5Gy with HLU / Sham with HLU	1.2160	0.9863	1.4993	0.0657	0.1478
**IL_1beta**	**1.5Gy with HLU / Sham with HLU**	**1.2681**	**1.0688**	**1.5045**	**0.0087**	**0.0335**
**IL_2**	**1.5Gy with HLU / Sham with HLU**	**1.4533**	**1.1669**	**1.8102**	**0.0019**	**0.0169**
**IL_4**	**1.5Gy with HLU / Sham with HLU**	**1.1931**	**1.0345**	**1.3760**	**0.0176**	**0.0475**

outcome	contrast	ratio	lower.CL	upper.CL	p.value	q
IL 5	1.5Gy with HLU / Sham with HLU	1.0460	0.9349	1.1703	0.4146	0.5316
IL 6	1.5Gy with HLU / Sham with HLU	1.1650	0.9339	1.4533	0.1661	0.2491
**IL_10**	**1.5Gy with HLU / Sham with HLU**	**1.3043**	**1.0711**	**1.5884**	**0.0105**	**0.0344**
IL_12p70	1.5Gy with HLU / Sham with HLU	0.9785	0.7994	1.1978	0.8258	0.8575
IL 13	1.5Gy with HLU / Sham with HLU	1.0704	0.8461	1.3543	0.5544	0.6508
IL 17A	1.5Gy with HLU / Sham with HLU	1.0896	0.8719	1.3617	0.4332	0.5316
**IL_18**	**1.5Gy with HLU / Sham with HLU**	**1.3613**	**1.1095**	**1.6703**	**0.0049**	**0.0276**
**IP_10**	**1.5Gy with HLU / Sham with HLU**	**1.1002**	**1.0240**	**1.1821**	**0.0115**	**0.0344**
Leptin	1.5Gy with HLU / Sham with HLU	1.1887	0.9676	1.4602	0.0955	0.1983
LIX	1.5Gy with HLU / Sham with HLU	1.0174	0.7315	1.4150	0.9148	0.9148
MCP_ 1	1.5Gy with HLU / Sham with HLU	1.0269	0.8753	1.2049	0.7335	0.7922
**MIP_1alpha**	**1.5Gy with HLU / Sham with HLU**	**1.3520**	**1.1393**	**1.6044**	**0.0014**	**0.0169**
**MIP** 2	1.5Gy with HLU / Sham with HLU	1.1373	1.0021	1.2908	0.0466	0.1145
RANTES	1.5Gy with HLU / Sham with HLU	1.0647	0.9368	1.2101	0.3206	0.4329
TNFalpha	1.5Gy with HLU / Sham with HLU	1.1461	0.9432	1.3926	0.1606	0.2491
**VEGF**	**1.5Gy with HLU / Sham with HLU**	**1.9875**	**1.2572**	**3.1420**	**0.0051**	**0.0276**

Between group comparison

A mixed effects model with random intercept was fitted to the log-transformed value of each cytokine as the outcome variable. Fixed effects for sampling time and study group were included. Pairwise comparisons between the groups were performed, with no adjustment for multiple testing. Significant differences are shown in bold.

Benjamini & Hochberg method ("fdr") was used for p value adjustment between cytokines.
