# Supplementary material for: Multiple simulated spaceflight stressors impact cardiac fibrosis, calcium dynamics, immune function, cytokines and gene variants in rat, Rattus norvegicus
Source: PLoS One. 2026 Sep 30;21(9):e0357485. doi: 10.1371/journal.pone.0357485 (PMC13626364; doi:10.1371/journal.pone.0357485)
Supplement: Supplementary file 9 [file pone.0357485.s009.pdf]

Gene	Group	impact	variant type	hgvs.c	hgvs.p	VAF%
Asxl1	Sham unique	HIGH	frameshift	c.1607delG	p.Arg536fs	0.917
Asxl1	Sham unique	HIGH	frameshift	c.1611_1614delAGTC	p.Arg537fs	0.923
Asxl1	Sham unique	HIGH	frameshift	c.1620delG	p.Pro541fs	0.933
Asxl1	Sham unique	HIGH	frameshift	c.1623delG	p.His542fs	0.933
Asxl1	Sham unique	HIGH	frameshift	c.1627_1628delGG	p.Gly543fs	0.933
Asxl1	Sham unique	HIGH	splice donor	c.1632+2delT		0.944
Asxl1	Sham unique	LOW	splice region	c.1632+6T>G		0.947
Smc3	Sham unique	LOW	splice region	c.2644+6T>A		0.143
Smc3	Sham unique	LOW	splice region	c.2644+6_2644+7insTGACAAAACAGAA		0.282
Smc3	Sham unique	LOW	splice region	c.2644+4_2644+5insTTCC		0.293
Smc3	Sham unique	HIGH	splice donor	c.2644+1_2644+2insGA		0.381
Smc3	Sham unique	HIGH	splice donor	c.2644_2644+1insATTT		0.39
Smc3	Sham unique	LOW	splice region	c.2644+2_2644+3insTTGGATAATTCCATTGACAAAACAGAAGCTGGAATTAAAGAGCTCCAG		0.365; 0.332
Smc3	Sham unique	HIGH	splice donor	c.2644+1G>A		0.365; 0.338
Smc3	IR unique	LOW	splice region	c.3297+3_3297+4insTCATTTACAGGAAAGCAAGGCGAGATGAGAGAAATGCAGCAGCTTTCAGGAGGGCAG		0.408
Smc3	Sham unique	LOW	splice region	c.3582+5_3582+6insCGACGTGATCACAGCAAAGATGGCCAAAGAC		0.153
Smc3	Sham unique	LOW	splice region	c.3582+3_3582+4insGTCAC		0.155
Smc3	Sham unique	HIGH	splice donor	c.3582+1_3582+2insT		0.161
Suz12	Sham unique	LOW	splice region	c.912+4_912+5delAA		0.143; 0.286; 0.91
Suz12	IR unique	LOW	splice region	c.912+4delA		0.959; 0.964; 0.964
Pds5b	Sham unique	LOW	splice region	c.3530-4_3530-3insA		0.917
Pds5b	Sham unique	HIGH	frameshift	c.3530-1_3530insC	p.Asp1177fs	0.923
Pds5b	Sham unique	HIGH	frameshift	c.3530_3531insC	p.Glu1178fs	0.923
Pds5b	Sham unique	HIGH	frameshift	c.3537dupG	p.Asn1180fs	0.933
Pds5b	Sham unique	HIGH	frameshift	c.3538_3539insCC	p.Asn1180fs	0.941
Pds5b	Sham unique	HIGH	frameshift	c.3545delC	p.Ala1182fs	0.947
Pds5b	Sham unique	HIGH	frameshift	c.3549_3550insT	p.Glu1184fs	0.948
Brcc3	Sham unique	HIGH	frameshift	c.281_282insTGTTGAGGATGTGGAGGTAAGCAAAATTGTAGAACCACTTTGGAAATCAGTTTGTTAGCTTCTAAGAAAATTGGGAGTAG	p.Glu95fs	0.36
Brcc3	IR unique	MODERATE	conservative inframe	c.283_284insGTAAGCAAAATTGTAGAACCACTTTGGAAATCAGTTTGTTAGCTTCTAAGAAAATTGGGAGTAGAG	p.Pro94_Glu95insGlyLysGlnAsnCysArgThrThrLeuGluIleSerLeuLeuAlaSerLysLysIleGlySerArg	0.234
Ep300	Sham unique	MODERATE	missense	c.1152G>T	p.Gln384His	0.19
Ep300	Sham unique	MODERATE	missense	c.1168C>A	p.Gln390Lys	0.143
Ep300	Sham unique	LOW	splice region	c.1171+8G>T		0.169
Ep300	Sham unique	LOW	splice region	c.1171+7A>T		0.182
Ep300	Sham unique	LOW	splice region	c.1171+4A>T		0.279; 0.14
Ep300	IR unique	HIGH	frameshift	c.2145_2146delGA	p.Trp715fs	0.743
Ep300	IR unique	HIGH	stop gained	c.2145G>A	p.Trp715*	0.142
Cblb	IR unique	MODERATE	missense	c.2505T>A	p.His835Gln	0.153
Cux1	Sham unique	HIGH	splice donor	c.306+1_306+2insAGCGGCAGAGAAAATACCTGGGCCTGAGCCCCTGGGACAAGGCCACACTTGGCATGGGGCGTCTGATTCTCTCCAACAAAA		0.092; 0.143
Crebbp	Sham unique	MODERATE	conservative inframe	c.4687_4689delGAA	p.Glu1563del	0.102
Pdss2	Sham unique	MODERATE	missense	c.714T>A	p.Asn238Lys	0.11
Pdss2	Sham unique	MODERATE	missense	c.719T>A	p.Ile240Asn	0.121
Prpf8	Sham unique	MODERATE	missense	c.3072T>A	p.His1024Gln	0.081
Prpf8	Sham unique	HIGH	splice donor	c.4946+1G>T		0.178
Idh2	IR unique	LOW	splice region	c.968-3_968-2insCCCCCC		0.875
Idh2	Sham unique	LOW	splice region	c.968-3_968-2insCCCC		0.884; 0.885; 0.243
Csf1r	Sham unique	LOW	splice region	c.1879+3_1879+4delGA		0.154
Dnmt3a	Sham unique	LOW	splice region	c.2161+8_2161+10delTCC		0.094
U2af2	IR unique	LOW	splice region	c.186-4T>A		0.129

Supplement Table 2
