# Supplementary material for: Multiple simulated spaceflight stressors impact cardiac fibrosis, calcium dynamics, immune function, cytokines and gene variants in rat, Rattus norvegicus
Source: PLoS One. 2026 Sep 30;21(9):e0357485. doi: 10.1371/journal.pone.0357485 (PMC13626364; doi:10.1371/journal.pone.0357485)
Supplement: Supplementary file 10 [file pone.0357485.s010.pdf]

Gene	Group	impact	variant type	hgvs.c	hgvs.p	ENST	chromosome	position	reference	alternative
Asxl1	Sham unique	HIGH	frameshift_variant	c.1607delG	p.Arg536fs	ENSRNOT00000091808.1	3	148896365	AG	A
Asxl1	Sham unique	HIGH	frameshift_variant	c.1611_1614delAGTC	p.Arg537fs	ENSRNOT00000091808.1	3	148896369	GAGTC	G
Asxl1	Sham unique	HIGH	frameshift_variant	c.1620delG	p.Pro541fs	ENSRNOT00000091808.1	3	148896377	CG	C
Asxl1	Sham unique	HIGH	frameshift_variant	c.1623delG	p.His542fs	ENSRNOT00000091808.1	3	148896381	CG	C
Asxl1	Sham unique	HIGH	frameshift_variant	c.1627_1628delGG	p.Gly543fs	ENSRNOT00000091808.1	3	148896385	TGG	T
Asxl1	Sham unique	HIGH	splice_donor_variant&intron_variant	c.1632+2delT		ENSRNOT00000091808.1	3	148896392	GT	G
Asxl1	Sham unique	LOW	splice_region_variant&intron_variant	c.1632+6T>G		ENSRNOT00000091808.1	3	148896397	T	G
Asxl1	Shared	HIGH	frameshift_variant&splice_region_variant	c.1096delC	p.Val367fs	ENSRNOT00000091808.1	3	148895806	GC	G
Asxl1	Shared	HIGH	splice_acceptor_variant&splice_region_va	c.1099-1delG		ENSRNOT00000091808.1	3	148895814	TG	T
Asxl1	Shared	HIGH	splice_donor_variant&splice_region_varia	c.1134+2delC		ENSRNOT00000091808.1	3	148895852	AC	A
Asxl1	Shared	HIGH	frameshift_variant	c.1153delG	p.Ala385fs	ENSRNOT00000091808.1	3	148895870	CG	C
Asxl1	Shared	HIGH	splice_donor_variant&splice_region_varia	c.1158+1_1158+2insA		ENSRNOT00000091808.1	3	148895880	T	TA
Asxl1	Shared	HIGH	frameshift_variant&splice_region_variant	c.1160_1161delAC	p.Asn387fs	ENSRNOT00000091808.1	3	148895885	AAC	A
Asxl1	Shared	HIGH	splice_donor_variant&splice_region_varia	c.1257+2delA		ENSRNOT00000091808.1	3	148895984	CA	C
Asxl1	Shared	MODERATE	missense_variant	c.1541T>G	p.Leu514Arg	ENSRNOT00000091808.1	3	148896271	T	G
Asxl1	Shared	HIGH	frameshift_variant&splice_region_variant	c.1559_1560insCT	p.Arg521fs	ENSRNOT00000091808.1	3	148896289	C	CCT
Asxl1	Shared	MODERATE	disruptive_inframe_deletion&splice_regi	c.1577_1582delTTGTCG	p.Val526_Val527del	ENSRNOT00000091808.1	3	148896306	AGTTGTC	A
Asxl1	Shared	MODERATE	missense_variant&splice_region_variant	c.1582G>A	p.Ala528Thr	ENSRNOT00000091808.1	3	148896313	G	A
Asxl1	Shared	LOW	splice_region_variant&intron_variant	c.1582+4_1582+7delTGTT		ENSRNOT00000091808.1	3	148896316	GTGTT	G
Asxl1	Shared	HIGH	frameshift_variant	c.1586_1587delGC	p.Arg529fs	ENSRNOT00000091808.1	3	148896343	CCG	C
Bcor	Shared	LOW	splice_region_variant&intron_variant	c.3061-3T>C		ENSRNOT00000041003.4	X	11673206	T	C
Brcc3	IR unique	MODERATE	conservative_inframe_insertion	c.283_284insGTAAGCAAAATTGT	p.Pro94_Glu95insGlyLysGlnAsnC	ENSRNOT00000071068.2	9	9865623	T	TCTCTACTCCCAATTTTCTTAGAAGCTAACAAACTGATTTCCAAAGTGGTTCTACAATTTGCTTTAC
Brcc3	Sham unique	HIGH	frameshift_variant&stop_gained	c.281_282insTGTTGAGGATGTGG	p.Glu95fs	ENSRNOT00000071068.2	9	9865625	T	TCTACTCCCAATTTTCTTAGAAGCTAACAAACTGATTTCCAAAGTGGTTCTACAATTTGCTTACCTCCCACATCCTCAAGCA
Brcc3	Shared	MODERATE	missense_variant	c.764G>T	p.Ser255Ile	ENSRNOT00000071068.2	9	9865143	C	A
Brcc3	Shared	HIGH	stop_gained	c.755C>A	p.Ser252*	ENSRNOT00000071068.2	9	9865152	G	T
Brcc3	Shared	MODERATE	missense_variant	c.660C>A	p.Asp220Glu	ENSRNOT00000071068.2	9	9865247	G	T
Brcc3	Shared	MODERATE	missense_variant	c.338G>A	p.Arg113His	ENSRNOT00000071068.2	9	9865569	C	T
Brcc3	Shared	MODERATE	missense_variant	c.323C>T	p.Ala108Val	ENSRNOT00000071068.2	9	9865584	G	A
Brcc3	Shared	MODERATE	missense_variant	c.302C>G	p.Ser101Trp	ENSRNOT00000071068.2	9	9865605	G	C
Cblb	IR unique	MODERATE	missense_variant	c.2505T>A	p.His835Gln	ENSRNOT00000077220.1	11	51054655	A	T
Cblb	Shared	LOW	splice_region_variant&intron_variant	c.2429-5T>C		ENSRNOT00000077220.1	11	51054736	A	G
Cblb	Shared	MODERATE	missense_variant	c.1735G>A	p.Gly579Ser	ENSRNOT00000077220.1	11	51076138	C	T
Cnt4bp	Sham unique	MODERATE	conservative_inframe_deletion	c.4687_4689delGAA	p.Glu1563del	ENSRNOT00000057079.5	10	11712085	CAAG	C
Cnt4bp	Shared	MODERATE	disruptive_inframe_deletion	c.6639_6641delGCA	p.Gln2214del	ENSRNOT00000057079.5	10	11717565	ACAG	A
Csf3r	Sham unique	LOW	splice_region_variant&intron_variant	c.1879+3_1879+4delGA		ENSRNOT00000049357.4	18	56451411	TGA	T
Csf3r	Shared	MODERATE	missense_variant	c.2239A>G	p.Ser747Gly	ENSRNOT00000011694.6	5	144050006	A	G
Csf3r	Shared	MODERATE	missense_variant	c.2422A>G	p.Ser808Gly	ENSRNOT00000011694.6	5	144050189	A	G
Cux1	Sham unique	HIGH	splice_donor_variant&intron_variant	c.306+1_306+2insAGCGGCAGAGAAAATACCTGGGCCTGAGCCCCTGGG		ENSRNOT00000044841.6	12	23466295	G	GAGCGGCAGAGAAAATACCTGGGCCTGAGCCCCTGGGACAAGGCCACACTTGGCATGGGGCGTCTGATTCTCTCCAACAAAA
Cux1	Shared	MODERATE	missense_variant	c.275G>A	p.Arg92His	ENSRNOT00000044841.6	12	23466263	G	A
Dnmt3a	Sham unique	LOW	splice_region_variant&intron_variant	c.2161+8_2161+10delTTC		ENSRNOT00000047210.3	6	28334811	TTCC	T
Ep300	IR unique	HIGH	frameshift_variant	c.2145_2146delGA	p.Trp715fs	ENSRNOT00000006206.7	7	122861055	GGA	G
Ep300	IR unique	HIGH	stop_gained	c.2145G>A	p.Trp715*	ENSRNOT00000006206.7	7	122861056	G	A
Ep300	Sham unique	MODERATE	missense_variant	c.1152G>T	p.Gln384His	ENSRNOT00000006206.7	7	122846556	G	T
Ep300	Sham unique	MODERATE	missense_variant	c.1168C>A	p.Gln390Lys	ENSRNOT00000006206.7	7	122846572	C	A
Ep300	Sham unique	LOW	splice_region_variant&intron_variant	c.1171+4A>T		ENSRNOT00000006206.7	7	122846579	A	T
Ep300	Sham unique	LOW	splice_region_variant&intron_variant	c.1171+7A>T		ENSRNOT00000006206.7	7	122846582	A	T
Ep300	Sham unique	LOW	splice_region_variant&intron_variant	c.1171+8G>T		ENSRNOT00000006206.7	7	122846583	G	T
Ep300	Shared	HIGH	frameshift_variant	c.2145delG	p.Trp715fs	ENSRNOT00000006206.7	7	122861056	TG	T
Flt3	Shared	MODERATE	conservative_inframe_deletion	c.1816_1821delGCGTTT	p.Ala606_Phe607del	ENSRNOT00000088957.1	12	9415017	AGTTTGC	A
Flt3	Shared	LOW	splice_region_variant&intron_variant	c.1843+8A>T		ENSRNOT00000088957.1	12	9415057	A	T
Flt3	Shared	LOW	splice_region_variant&intron_variant	c.1844-5G>T		ENSRNOT00000088957.1	12	9415130	G	T
Flt3	Shared	HIGH	frameshift_variant	c.1855delC	p.Ser620fs	ENSRNOT00000088957.1	12	9415149	TC	T
Gnas	Shared	MODERATE	missense_variant	c.656T>G	p.Leu219Arg	ENSRNOT00000090516.5	3	172370952	T	G
Gnas	Shared	HIGH	frameshift_variant&splice_region_variant	c.2125_2126insA	p.Pro709fs	ENSRNOT00000090989.1	3	172387799	C	CA
Gnas	Shared	HIGH	splice_acceptor_variant&splice_region_va	c.28-1delC		ENSRNOT00000071215.2	3	172420620	GC	G
Gnas	Shared	HIGH	frameshift_variant&splice_region_variant	c.103delA	p.Ser35fs	ENSRNOT00000071215.2	3	172420696	GA	G
Gnas	Shared	HIGH	splice_donor_variant&splice_region_varia	c.105+1delC		ENSRNOT00000071215.2	3	172420699	TC	T
Gnas	Shared	HIGH	frameshift_variant	c.109dupG	p.Ala37fs	ENSRNOT00000071215.2	3	172420705	C	CG
Gnas	Shared	LOW	splice_region_variant&intron_variant	c.2505-7delG		ENSRNOT00000090989.1	3	172433136	AG	A
Gnas	Shared	HIGH	frameshift_variant	c.2511delA	p.Gly838fs	ENSRNOT00000090989.1	3	172433147	GA	G
Gnas	Shared	HIGH	frameshift_variant	c.2516_2517delCC	p.Pro839fs	ENSRNOT00000090989.1	3	172433152	GCC	G
Gnas	Shared	HIGH	frameshift_variant&splice_region_variant	c.2526delT	p.Gln843fs	ENSRNOT00000090989.1	3	172433163	GT	G
Gnas	Shared	HIGH	frameshift_variant&splice_region_variant	c.2522delT	p.Val842fs	ENSRNOT00000090989.1	3	172433171	AT	A
Gnas	Shared	HIGH	frameshift_variant	c.2530delG	p.Val846fs	ENSRNOT00000090989.1	3	172433324	TG	T
Gnas	Shared	HIGH	frameshift_variant	c.2585_2586insCC	p.Leu863fs	ENSRNOT00000090989.1	3	172433329	G	GCC
Gnas	Shared	HIGH	splice_acceptor_variant&splice_donor_va	c.2622+1delT		ENSRNOT00000090989.1	3	172433360	GT	G
Gnas	Shared	HIGH	frameshift_variant	c.382delG	p.Ala128fs	ENSRNOT00000071215.2	3	172433379	TG	T
Gnas	Shared	HIGH	frameshift_variant	c.392_393delTT	p.Val131fs	ENSRNOT00000071215.2	3	172433389	GTT	G
Gnas	Shared	HIGH	splice_acceptor_variant&splice_donor_va	c.2664+1_2665-1delTT		ENSRNOT00000090989.1	3	172433406	CTT	C
Gnas	Shared	HIGH	frameshift_variant&splice_region_variant	c.2677dupC	p.Leu893fs	ENSRNOT00000090989.1	3	172433420	A	AC
Gnas	Shared	HIGH	frameshift_variant	c.423delC	p.Phe142fs	ENSRNOT00000071215.2	3	172433535	GC	G
Gnas	Shared	HIGH	frameshift_variant	c.431delG	p.Arg144fs	ENSRNOT00000071215.2	3	172433544	CG	C
Gnas	Shared	HIGH	frameshift_variant	c.461delA	p.His154fs	ENSRNOT00000071215.2	3	172433574	CA	C
Gnas	Shared	HIGH	frameshift_variant	c.466delA	p.Thr156fs	ENSRNOT00000071215.2	3	172433578	GA	G
Gnas	Shared	MODERATE	missense_variant	c.501A>T	p.Lys167Asn	ENSRNOT00000071215.2	3	172433615	A	T
Gnas	Shared	HIGH	frameshift_variant	c.508delT	p.Trp170fs	ENSRNOT00000071215.2	3	172433620	AT	A
Gnas	Shared	HIGH	frameshift_variant	c.536delA	p.Asn179fs	ENSRNOT00000071215.2	3	172433646	CA	C
Gnas	Shared	HIGH	frameshift_variant	c.546delA	p.Asp183fs	ENSRNOT00000071215.2	3	172433681	CA	C
Gnas	Shared	HIGH	frameshift_variant	c.572_573delCC	p.Pro191fs	ENSRNOT00000071215.2	3	172433707	GCC	G
Gnas	Shared	HIGH	frameshift_variant	c.634_635insG	p.His212fs	ENSRNOT00000071215.2	3	172433771	C	CG
Gnas	Shared	HIGH	frameshift_variant	c.654_655insA	p.Trp219fs	ENSRNOT00000071215.2	3	172433791	G	GA
Gnas	Shared	HIGH	frameshift_variant	c.657dupG	p.Thr220fs	ENSRNOT00000071215.2	3	172433792	T	TG
Gnas	Shared	HIGH	frameshift_variant	c.671delA	p.Asn224fs	ENSRNOT00000071215.2	3	172433804	GA	G
Idh2	IR unique	LOW	splice_region_variant&intron_variant	c.968-3_968-2insCCCCCC		ENSRNOT00000019059.5	1	141875674	T	TGGGGGG
Idh2	Sham unique	LOW	splice_region_variant&intron_variant	c.968-3_968-2insCCCC		ENSRNOT00000019059.5	1	141875674	T	TGGGGG
Jak1	Shared	MODERATE	missense_variant	c.425A>G	p.Lys142Arg	ENSRNOT00000064542.1	5	120016767	T	C
Jak3	Shared	MODERATE	missense_variant	c.989C>G	p.Pro330Arg	ENSRNOT00000025312.6	16	20112467	C	G
Kras	Shared	HIGH	splice_donor_variant&intron_variant	c.-13+1_-13+2insG		ENSRNOT00000012887.6	4	179515438	A	AC
Pds5b	Sham unique	LOW	splice_region_variant&intron_variant	c.3530-4_3530-3insA		ENSRNOT00000050733.6	12	783241	G	GA
Pds5b	Sham unique	HIGH	frameshift_variant&splice_region_variant	c.3530-1_3530insC	p.Asp1177fs	ENSRNOT00000050733.6	12	783244	G	GC
Pds5b	Sham unique	HIGH	frameshift_variant&splice_region_variant	c.3530_3531insC	p.Glu1178fs	ENSRNOT00000050733.6	12	783245	A	AC
Pds5b	Sham unique	HIGH	frameshift_variant	c.3537dupG	p.Asn1180fs	ENSRNOT00000050733.6	12	783251	T	TG
Pds5b	Sham unique	HIGH	frameshift_variant	c.3538_3539insCC	p.Asn1180fs	ENSRNOT00000050733.6	12	783253	A	ACC
Pds5b	Sham unique	HIGH	frameshift_variant	c.3545delC	p.Ala1182fs	ENSRNOT00000050733.6	12	783259	GC	G
Pds5b	Sham unique	HIGH	frameshift_variant&stop_gained	c.3549_3550insT	p.Glu1184fs	ENSRNOT00000050733.6	12	783264	A	AT
Pds5b	Shared	HIGH	frameshift_variant	c.3565_3566insC	p.Phe1189fs	ENSRNOT00000050733.6	12	783280	T	TC
Pds5b	Shared	HIGH	frameshift_variant	c.3570_3571insCC	p.Leu1191fs	ENSRNOT00000050733.6	12	783284	A	ACC
Pds5b	Shared	HIGH	frameshift_variant	c.3593_3594insG	p.Glu1199fs	ENSRNOT00000050733.6	12	783308	A	AG
Pds5b	Shared	HIGH	frameshift_variant	c.3610delC	p.Val1205fs	ENSRNOT00000050733.6	12	783322	AC	A
Pdss2	Sham unique	MODERATE	missense_variant	c.714T>A	p.Asn238Lys	ENSRNOT00000067169.2	20	48142276	T	A
Pdss2	Sham unique	MODERATE	missense_variant	c.719T>A	p.Ile240Asn	ENSRNOT00000067169.2	20	48142281	T	A
Phip	Shared	LOW	splice_region_variant&intron_variant	c.3465-3_3465-2insTTTC		ENSRNOT00000011864.6	8	90407525	T	TGAAA
Phip	Shared	LOW	splice_region_variant&intron_variant	c.1197+8C>A		ENSRNOT00000011864.6	8	90451518	G	T
Phip	Shared	MODERATE	missense_variant	c.1181G>T	p.Arg394Leu	ENSRNOT00000011864.6	8	90451542	C	A
Phip	Shared	MODERATE	missense_variant	c.1179C>A	p.His393Gln	ENSRNOT00000011864.6	8	90451544	G	T
Phip	Shared	HIGH	splice_acceptor_variant&splice_region_va	c.1174-2delC		ENSRNOT00000011864.6	8	90451550	AG	A
Phip	Shared	HIGH	splice_donor_variant&splice_region_varia	c.1173+2C>A		ENSRNOT00000011864.6	8	90451553	G	T
Phip	Shared	MODERATE	missense_variant	c.1163G>T	p.Trp388Leu	ENSRNOT00000011864.6	8	90451565	C	A
Phip	Shared	MODERATE	disruptive_inframe_deletion	c.1149_1151delGCC	p.Gln383_Pro384delinsHis	ENSRNOT00000011864.6	8	90451576	GGGC	G
Phip	Shared	HIGH	frameshift_variant	c.1147_1148insTG	p.Gln383fs	ENSRNOT00000011864.6	8	90451580	T	TCA
Phip	Shared	MODERATE	missense_variant	c.1147C>A	p.Gln383Lys	ENSRNOT00000011864.6	8	90451581	G	T
Phip	Shared	MODERATE	missense_variant	c.1135C>G	p.Pro379Ala	ENSRNOT00000011864.6	8	90451593	G	C
Phip	Shared	MODERATE	missense_variant	c.1132C>A	p.Pro378Thr	ENSRNOT00000011864.6	8	90451596	G	T
Phip	Shared	HIGH	frameshift_variant&splice_region_variant	c.1128delT	p.Phe376fs	ENSRNOT00000011864.6	8	90451599	TA	T
Phip	Shared	HIGH	frameshift_variant&splice_donor_variant	c.1119_1126-9delGTCTTCACCAC	p.Glu373fs	ENSRNOT00000011864.6	8	90451610	GGGCCGCAG	G
Prpf8	Sham unique	MODERATE	missense_variant	c.3072T>A	p.His1024Gln	ENSRNOT00000005016.5	10	63643739	T	A
Prpf8	Sham unique	HIGH	splice_donor_variant&intron_variant	c.4946+1G>T		ENSRNOT00000005016.5	10	63651438	G	T
Setbp1	Shared	HIGH	frameshift_variant	c.3880_3881delGG	p.Gly1294fs	ENSRNOT00000021717.7	18	75095636	TCC	T
Setbp1	Shared	HIGH	frameshift_variant	c.3848dupC	p.Pro1284fs	ENSRNOT00000021717.7	18	75095669	T	TG
Setbp1	Shared	HIGH	frameshift_variant	c.3827_3828insC	p.Gln1278fs	ENSRNOT00000021717.7	18	75095690	C	CG
Setbp1	Shared	HIGH	frameshift_variant	c.3784delA	p.Arg1262fs	ENSRNOT00000021717.7	18	75095733	CT	C
Setbp1	Shared	HIGH	frameshift_variant	c.3779dupA	p.Arg1261fs	ENSRNOT00000021717.7	18	75095738	C	CT
Setbp1	Shared	HIGH	frameshift_variant	c.3747dupC	p.Gly1250fs	ENSRNOT00000021717.7	18	75095770	C	CG
Setbp1	Shared	HIGH	frameshift_variant	c.3682dupA	p.Arg1228fs	ENSRNOT00000021717.7	18	75095835	C	CT
Setd2	Shared	MODERATE	missense_variant	c.485T>C	p.Val162Ala	ENSRNOT00000087154.1	8	118822162	T	C
Setd2	Shared	MODERATE	missense_variant	c.488C>T	p.Ala163Val	ENSRNOT00000087154.1	8	118822165	C	T
Setd2	Shared	MODERATE	missense_variant	c.659C>T	p.Ala220Val	ENSRNOT00000087154.1	8	118822336	C	T
Setd2	Shared	MODERATE	disruptive_inframe_insertion	c.2374_2375insCTGAACCTTCAGT	p.Asn791_Arg792insThrGluProSe	ENSRNOT00000087154.1	8	118824049	A	ATACTGAACCTTCAGT
Setd2	Shared	MODERATE	missense_variant	c.3421G>C	p.Val1141Leu	ENSRNOT00000087154.1	8	118825098	G	C
Setd2	Shared	MODERATE	missense_variant	c.3448A>G	p.Ile1150Val	ENSRNOT00000087154.1	8	118825125	A	G
Setd2	Shared	MODERATE	missense_variant	c.3461T>C	p.Met1154Thr	ENSRNOT00000087154.1	8	118825138	T	C
Setd2	Shared	LOW	splice_region_variant&intron_variant	c.4596-7T>C		ENSRNOT00000087154.1	8	118834393	T	C
Setd2	Shared	LOW	splice_region_variant&intron_variant	c.6840+8A>G		ENSRNOT00000087154.1	8	118864275	A	G
Setd2	Shared	LOW	splice_region_variant&intron_variant	c.6975+8G>A		ENSRNOT00000087154.1	8	118868332	G	A
Sf1	Shared	MODERATE	disruptive_inframe_deletion	c.1517_1519delAGC	p.Gln506del	ENSRNOT00000028628.7	1	221746839	GGCA	G
Smc1a	Shared	HIGH	frameshift_variant	c.3204dupC	p.Asp1069fs	ENSRNOT00000004364.7	X	21752784	A	AC
Smc1a	Shared	HIGH	frameshift_variant	c.3223delG	p.Glu1075fs	ENSRNOT00000004364.7	X	21752802	TG	T
Smc1a	Shared	HIGH	splice_acceptor_variant&splice_donor_va	c.3237+1delA		ENSRNOT00000004364.7	X	21752818	CA	C
Smc1a	Shared	HIGH	frameshift_variant	c.3251delA	p.Asn1084fs	ENSRNOT00000004364.7	X	21752831	GA	G
Smc1a	Shared	LOW	splice_region_variant&intron_variant	c.3260+6delA		ENSRNOT00000004364.7	X	21752847	GA	G
Smc1a	Shared	LOW	splice_region_variant&intron_variant	c.3260+8delC		ENSRNOT00000004364.7	X	21752849	TC	T
Smc1a	Shared	LOW	splice_region_variant&intron_variant	c.3261-4_3261-3insC		ENSRNOT00000004364.7	X	21752956	T	TC
Smc1a	Shared	HIGH	frameshift_variant	c.3277_3278insC	p.Phe1093fs	ENSRNOT00000004364.7	X	21752976	T	TC
Smc1a	Shared	HIGH	frameshift_variant	c.3273_3274insC	p.Leu1092fs	ENSRNOT00000004364.7	X	21752978	T	TC
Smc1a	Shared	LOW	splice_region_variant&intron_variant	c.3434+6_3434+7insC		ENSRNOT00000004364.7	X	21753142	G	GC
Smc3	IR unique	LOW	splice_region_variant&intron_variant	c.3297+3_3297+4insTCATTTACAGGAAAGCAAGGCGAGATGAGAGAA		ENSRNOT00000019560.7	1	274350266	A	ATCATTTACAGGAAAGCAAGGCGAGATGAGAGAAATGCAGCAGCTTTCAGGAGG
Smc3	Sham unique	HIGH	splice_donor_variant&intron_variant	c.2644_2644+1insATTT		ENSRNOT00000019560.7	1	274348040	G	GATTT
Smc3	Sham unique	HIGH	splice_donor_variant&intron_variant	c.2644+1G>A		ENSRNOT00000019560.7	1	274348041	G	A
Smc3	Sham unique	HIGH	splice_donor_variant&intron_variant	c.2644+1_2644+2insGA		ENSRNOT00000019560.7	1	274348041	G	GGA
Smc3	Sham unique	LOW	splice_region_variant&intron_variant	c.2644+2_2644+3insTTGGATAATTCCATTGACAAAACAGAAGCTGGA		ENSRNOT00000019560.7	1	274348042	T	TTTGGATAATTCCATTGACAAAACAGAAGCTGGAATTAAAGAGCTCCAGAAAAGC
Smc3	Sham unique	LOW	splice_region_variant&intron_variant	c.2644+4_2644+5insTTCC		ENSRNOT00000019560.7	1	274348044	A	ATTCC
Smc3	Sham unique	LOW	splice_region_variant&intron_variant	c.2644+6T>A		ENSRNOT00000019560.7	1	274348046	T	A
Smc3	Sham unique	LOW	splice_region_variant&intron_variant	c.2644+6_2644+7insTGACAAAACAGAA		ENSRNOT00000019560.7	1	274348046	T	TTGACAAAACAGAA
Smc3	Sham unique	HIGH	splice_donor_variant&intron_variant	c.3582+1_3582+2insT		ENSRNOT00000019560.7	1	274351918	G	GT
Smc3	Sham unique	LOW	splice_region_variant&intron_variant	c.3582+3_3582+4insGTCAC		ENSRNOT00000019560.7	1	274351920	A	AGTCAC
Smc3	Sham unique	LOW	splice_region_variant&intron_variant	c.3582+5_3582+6insCGACGTGATCACAGCAAAGATGGCCAAAGAC		ENSRNOT00000019560.7	1	274351922	T	TCGACGTGATCACAGCAAAGATGGCCAAAGAC
Smc3	Shared	HIGH	frameshift_variant&splice_donor_variant	c.2891_2893-3delAGGTGAGTCTG	p.Gln964fs	ENSRNOT00000019560.7	1	274349580	CAGGTGAGTI	C
Smc3	Shared	MODERATE	missense_variant	c.2899C>T	p.Arg967Trp	ENSRNOT00000019560.7	1	274349675	C	T
Smc3	Shared	MODERATE	missense_variant	c.2914T>C	p.Cys972Arg	ENSRNOT00000019560.7	1	274349690	T	C
Smc3	Shared	MODERATE	missense_variant	c.3178G>A	p.Gly1060Arg	ENSRNOT00000019560.7	1	274350144	G	A
Smc3	Shared	LOW	splice_region_variant&intron_variant	c.3582+2_3582+3insTAGTCACATCGACGTGATCACAGCAAAGATGGC		ENSRNOT00000019560.7	1	274351919	T	TTAGTCACATCGACGTGATCACAGCAAAGATGGCCAAAGACTTTGTAGAAGACGA
Smc3	Shared	MODERATE	missense_variant	c.3610G>A	p.Glu1204Lys	ENSRNOT00000019560.7	1	274352443	G	A
Stag1	Shared	MODERATE	missense_variant	c.1873G>C	p.Glu625Gln	ENSRNOT00000079618.1	8	109253381	G	C
Stag1	Shared	MODERATE	missense_variant	c.3266C>T	p.Ala1089Val	ENSRNOT00000079618.1	8	109326167	C	T
Suz12	IR unique	LOW	splice_region_variant&intron_variant	c.912+4delA		ENSRNOT00000079002.1	10	67353453	TA	T
Suz12	Sham unique	LOW	splice_region_variant&intron_variant	c.912+4_912+5delAA		ENSRNOT00000079002.1	10	67353453	TAA	T
Suz12	Shared	MODERATE	disruptive_inframe_insertion	c.92_94dupCGG	p.Ala31dup	ENSRNOT00000079002.1	10	67325428	T	TCGG
Suz12	Shared	LOW	splice_region_variant&intron_variant	c.503-6A>G		ENSRNOT00000079002.1	10	67347492	A	G
Suz12	Shared	HIGH	frameshift_variant	c.960dupA	p.Phe321fs	ENSRNOT00000079002.1	10	67357145	T	TA
Suz12	Shared	HIGH	frameshift_variant	c.967delG	p.Glu323fs	ENSRNOT00000079002.1	10	67357154	AG	A
Suz12	Shared	HIGH	frameshift_variant	c.978delA	p.Glu327fs	ENSRNOT00000079002.1	10	67357162	GA	G
Suz12	Shared	HIGH	frameshift_variant	c.983delG	p.Gly328fs	ENSRNOT00000079002.1	10	67357169	AG	A
Suz12	Shared	HIGH	frameshift_variant	c.990delC	p.Leu331fs	ENSRNOT00000079002.1	10	67357175	TC	T
Suz12	Shared	MODERATE	missense_variant	c.1006A>T	p.Ile336Phe	ENSRNOT00000079002.1	10	67357194	A	T
Suz12	Shared	MODERATE	missense_variant	c.1009T>C	p.Ser337Pro	ENSRNOT00000079002.1	10	67357197	T	C
Suz12	Shared	LOW	splice_region_variant&intron_variant	c.1013+7delC		ENSRNOT00000079002.1	10	67357207	TC	T
Tet2	Shared	MODERATE	missense_variant	c.4481T>C	p.Met1494Thr	ENSRNOT00000046775.5	2	238723710	A	G
Tet2	Shared	MODERATE	missense_variant	c.2502G>T	p.Gln834His	ENSRNOT00000046775.5	2	238743634	C	A
Tet2	Shared	MODERATE	missense_variant	c.2417G>C	p.Arg806Thr	ENSRNOT00000046775.5	2	238743719	C	G
Tet2	Shared	MODERATE	missense_variant	c.2312A>T	p.Glu771Val	ENSRNOT00000046775.5	2	238743824	T	A
Tet2	Shared	MODERATE	missense_variant	c.2254C>A	p.Gln752Lys	ENSRNOT00000046775.5	2	238743882	G	T
Tet2	Shared	MODERATE	conservative_inframe_deletion	c.2209_2220delGAAAATCAGTAC	p.Glu737_Tyr740del	ENSRNOT00000046775.5	2	238743915	AGTACTGATI	A
Tet2	Shared	MODERATE	missense_variant	c.2179A>C	p.Thr727Pro	ENSRNOT00000046775.5	2	238743957	T	G
Tet2	Shared	MODERATE	missense_variant	c.1189C>T	p.Arg397Cys	ENSRNOT00000046775.5	2	238744947	G	A
Tet2	Shared	MODERATE	missense_variant	c.1111C>G	p.Arg371Gly	ENSRNOT00000046775.5	2	238745025	G	C
Tet2	Shared	MODERATE	missense_variant	c.823G>T	p.Ala275Ser	ENSRNOT00000046775.5	2	238745313	C	A
U2af2	IR unique	LOW	splice_region_variant&intron_variant	c.186-4T>A		ENSRNOT00000021391.6	1	72324725	A	T
Zrsr2	Shared	MODERATE	disruptive_inframe_deletion	c.1451_1468delATCGGAGCCGGA	p.Asn484_Arg489del	ENSRNOT00000045300.5	X	32327219	AAGCCGGAA	A

Supplement Table 3
