# Supplementary material for: Multiple simulated spaceflight stressors impact cardiac fibrosis, calcium dynamics, immune function, cytokines and gene variants in rat, Rattus norvegicus
Source: PLoS One. 2026 Sep 30;21(9):e0357485. doi: 10.1371/journal.pone.0357485 (PMC13626364; doi:10.1371/journal.pone.0357485)
Supplement: Supplementary file 11 [file pone.0357485.s011.pdf]

Supplement Table 4

Twins study		Rat study
Analyte	Approved HGNC Symbol	
BDNF	BDNF	
NGF	NGF	
EGF	EGF	EGF
ENA78	CXCL5	
FGFB	FGF2	
GMCSF	CSF2	GM-CSF
GROA	CXCL1	GRO/KC
HGF	HGF	
IFNA	IFNA2	
IFNB	IFNB1	
IFNG	IFNG	IFN gamma
IL10	IL10	IL-10
IL12P40	IL12B	
IL12P70	IL12 protein or IL12P70 is a heterodimer of IL12A and IL12B	
IL13	IL13	IL-13
IL15	IL15	
IL17A	IL17A	IL-17A
IL17F	IL17F	
IL18	IL18	IL-18
IL1A	IL1A	IL-1 alpha
IL1B	IL1B	IL-1 beta
IL2	IL2	IL-2
IL22	IL22	
IL23	IL23 protein is a heterodimer of IL23A and IL12B	
IL31	IL31	
IL5	IL5	IL-5
IL6	IL6	IL-6
IL7	IL7	
IL8	CXCL8	
IL9	IL9	
IP10	CXCL10	IP10
LEPTIN	LEP	Leptin
LIF	LIF	
MCSF	CSF1	
MCP1	CCL2	MCP-1
MCP3	CCL7	
PAI1	SERPINE1	
PDGFBB	Homodimer of PDGFB	
RESISTIN	RETN	
CD40L	CD40LG	
SCF	KITLG	
SDF1A	CXCL12	
ICAM1	ICAM1	
VCAM1	VCAM1	
TGFA	TGFA	
TNFA	TNF	TNFα
TNFB	LTA	
TRAIL	TNFSF10	
VEGF	VEGFA	VEGF-A
VEGFD	VEGFD	
		Eotaxin
		Fractalkine
		G-CSF
		IL-4
		IL-12p70
		MIP-1 alpha
		MIP-2
		RANTES
		LIX

Twins study - 17/50 (34%) of cytokines decreased after return to Earth + 190d.

Rat study - 20/27 (74%) of cytokines decreased after return to normal gravity + 30 - 270 d

Cytokines that decreased shown in blue.
